# *Clavibacter michiganensis* Reframed: The Story of How the Genomics Era Made a New Face for an Old Enemy

**DOI:** 10.1111/mpp.70093

**Published:** 2025-05-20

**Authors:** Ebrahim Osdaghi, Hamid Abachi, Marie‐Agnes Jacques

**Affiliations:** ^1^ Department of Plant Protection, College of Agriculture University of Tehran Karaj Iran; ^2^ Institut Agro, INRAE, IRHS, SFR QUASAV, CIRM‐CFBP Université d'Angers Angers France

**Keywords:** Actinobacteria, bacterial canker of tomato, Corynebacteria, *Microbacteriaceae*, quarantine pathogen, *Solanaceae*, *Solanum lycopersicum*

## Abstract

**Objective:**

Bacterial wilt and canker of tomato caused by the gram‐positive corynebacterial species *Clavibacter michiganensis* is an economically important disease threatening the tomato industry in both open‐air and greenhouse productions around the world. The disease occurs in many countries, with a particular importance in regions characterised by high temperature and water scarcity. Management of bacterial canker has been a major problem since its original description in 1909. This is due in part to the seedborne nature of the pathogen, allowing the bacterium to be transmitted over long distances via infected seeds, as well as a lack of effective treatment to clean seeds. Detection of the pathogen from seeds is difficult due to high competition on culture media with diverse members of the seed‐associated microbiota. Identification of the pathogen can also be difficult owing to the presence of different colony variants on culture media. In this review, we provide a historical perspective and an updated overview on the aetiology, epidemiology and management strategies of the bacterial canker disease. We also gathered recent molecular findings in the pathogenicity mechanisms and bioecology of *C. michiganensis* to boost management of the bacterial canker disease in the 21^st^ century tomato industry.

**Taxonomy:**

Class: *Actinobacteria*; Order: *Micrococcales*; Family: *Microbacteriaceae*; Genus: *Clavibacter*; Species: *Clavibacter michiganensis.*

**Disease Symptoms:**

Interveinal leaf chlorosis leading to necrotic areas. Canker on stems and lateral branches of the plant. Discolouration of vascular and pith tissues to dark yellow or brown. Small and early ripened fruits or discolouration of the placenta from white to yellow in the interior part of the ripening fruits.

**Host Range:**

Tomato ( *Solanum lycopersicum* ) is the main host of the pathogen while natural infection has also been reported on eggplant, pepper and wild nightshade plants.

**Synonyms (Historical/Non‐Preferred Scientific Names):**

*Aplanobacter michiganensis*; *Pseudomonas michiganense; Pseudomonas michiganensis; Bacterium michiganense*; *Phytomonas michiganensis; Mycobacterium michiganense; Erwinia michiganensis* (=*michiganense*); *Corynebacterium michiganense; Corynebacterium michiganense* pv. *michiganense*; *Corynebacterium michiganense* subsp. *michiganense; Clavibacter michiganensis* subsp. *michiganensis.*

**Microbiological Properties:**

The bacterium produces domed, round and shiny mucoid colonies on general culture media. Colonies are usually yellow‐pigmented, while pink‐pigmented strains are occasionally observed. Cells are gram‐positive, aerobic, non‐motile, non‐spore‐producing curved rods (coryneform).

**Distribution:**

Present in all continents.

**Phytosanitary Categorization:**

EPPO A2 List no. 50, EU 2019/2072 RNQP Annex IV. See EPPO (https://gd.eppo.int/taxon/CORBMI/categorization) and CABI (https://www.cabidigitallibrary.org/doi/10.1079/cabicompendium.15338) databases for further country‐specific categorisations. EPPO code: CORBMI.

## Graphical abstract

We provide a historical perspective and an updated overview on the aetiology, epidemiology and management strategies of tomato bacterial canker caused by *Clavibacter michiganensis.*

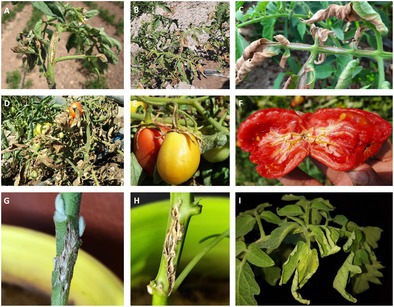

## 1 Taxonomic History of the Pathogen

In 1909, Erwin F. Smith observed a previously unreported disease on tomato in Grand Rapids, MI, United States. He named the disease ‘the Grand Rapids tomato disease’ and the causal agent *Bacterium michiganense*, which means a bacterium from Michigan (Smith 1910). The common name of the disease was changed to bacterial canker of tomato according to the characteristic canker symptoms on petioles and stems (Bryan 1930). Jensen (1934) noted that the tomato pathogen, along with the potato ring rot pathogen, was taxonomically similar to corynebacteria (Lehmann and Neumann 1896), thus proposed renaming the two species as *Corynebacterium michiganense* and *Corynebacterium sepedonicum* , respectively. *Corynebacterium michiganense* was applied to the tomato canker pathogen during the subsequent three decades.

During the 1970s, comprehensive chemotaxonomic investigations proposed the separation of plant‐pathogenic coryneform species from the original *Corynebacterium* members (Yamada and Komagata 1970; Jones 1975). For the first time, Dye and Kemp (1977) used pathovar designation in the classification of corynebacterial plant pathogens. They proposed classifying all coryneform plant‐pathogenic bacteria into four species within *Corynebacterium*, where the tomato canker pathogen, along with *Corynebacterium insidiosum* , *Corynebacterium nebraskense* , *Corynebacterium sepedonicum* and *Corynebacterium rathayi* , was designated as pathovars of *Corynebacterium michiganense* ; thus, the tomato canker pathogen was named *Corynebacterium michiganense* pv. *michiganense*. A few years later, the taxonomic status of the bacterial canker pathogen was changed from pathovar to subspecies level and named *Corynebacterium michiganense* subsp. *michiganense* (Carlson and Vidaver 1982).

In 1984, based on phenotypic features, for example, peptidoglycan content, Davis and his colleagues proposed the genus *Clavibacter* to include a newly described sugarcane pathogen ( *Clavibacter xyli* subsp. *xyli*), as well as the pathovars/subspecies of *Corynebacterium michiganense*. Therefore, the bacterial canker pathogen was renamed *Clavibacter michiganense* subsp. *michiganense* (Davis et al. 1984). However, under the nomenclature rules of bacterial taxonomy, the name was changed to *Clavibacter michiganensis* subsp. *michiganensis* in the subsequent years (Young et al. 1996). For the next three decades (1984–2014), all plant‐pathogenic members of *Clavibacter* were classified in one complex species *C. michiganense* with five subspecies reflecting their host of isolation and pathogenicity pattern (Vidaver and Davis 1988). All tomato‐associated *Clavibacter* strains were referred to as *C. michiganensis* subsp. *michiganensis* regardless of their pathogenicity, phenotypic features (e.g., colony pigmentation) and phylogenetic position (Jacques et al. 2012). The strains isolated from pepper plants were also classified as *C. michiganensis* subsp. *michiganensis* (Yim et al. 2012).

By the beginning of the genomics era, high‐throughput molecular techniques and DNA sequencing were used to decipher taxonomic relationships of *Clavibacter* lineages (Zaluga et al. 2011). Molecular phylogenetic analyses revealed higher taxonomic variations within *C. michiganensis* sensu lato strains than those previously reported on the basis of phenotypic data. For instance, Yim et al. (2012) noted that *C. michiganensis* subsp. *michiganensis* strains isolated from pepper had orange‐pigmented colonies with lower mucoidy than those of typical *C. michiganensis* subsp. *michiganensis* strains isolated from tomato. On the other hand, using phylogenetic analysis and polyphasic characterisation, Jacques et al. (2012) showed that nonpathogenic strains of *C. michiganensis* sensu lato originating from tomato were distinct from tomato‐pathogenic strains of the subspecies. In the subsequent years, tomato‐associated nonpathogenic members of *C. michiganensis* sensu lato were assigned to two new subspecies, *C. michiganensis* subsp. *californiensis* and *C. michiganensis* subsp. *chilensis* (Yasuhara‐Bell and Alvarez 2015). The pepper‐pathogenic members of the species were also assigned to a distinct subspecies, *C. michiganensis* subsp. *capsici* (Oh et al. 2016). Furthermore, nonpathogenic peach‐pigmented *Clavibacter* strains isolated from the tomato phyllosphere were shown to be distinct from all the above‐mentioned members of the genus (Osdaghi, Ansari, et al. 2018).

In 2018, reclassification of *Clavibacter* spp. into several new species was proposed based on genomic information, for example, average nucleotide identity (ANI) and digital DNA–DNA hybridisation (dDDH) indices (Li et al. 2018). The original subspecies of *C. michiganensis* sensu lato were elevated to the species level and the tomato bacterial canker pathogen was designated as *C. michiganensis* sensu stricto. Thus, unless otherwise stated, hereafter the tomato bacterial canker pathogen will be referred to as *C. michiganensis* in this text. Furthermore, nonpathogenic tomato‐associated members of *C. michiganensis* sensu lato were elevated to the species level where *C. michiganensis* subsp. *californiensis* was reclassified as *C. californiensis* and *C. michiganensis* subsp. *chilensis* was first transferred into *Clavibacter michiganensis* subsp. *phaseoli* (Osdaghi, Rahimi, et al. 2020) and then reclassified as *Clavibacter phaseoli* (Arizala et al. 2022). The pepper‐pathogenic subspecies *C. michiganensis* subsp. *capsici* was also designated as *Clavibacter capsici* (Li et al. 2018). More recently, peach‐pigmented nonpathogenic *Clavibacter* strains isolated from tomato were assigned to a new species as *Clavibacter lycopersici* (Osdaghi, Taghavi, et al. 2023).

In addition to the above‐mentioned four tomato‐associated *Clavibacter* lineages, phylogenomics and pathogenicity data showed that several hypothetical novel species could be identified within the genus, four of which were isolated from asymptomatic tomato tissues or seed lots (Osdaghi, Rahimi, et al. 2020; Yañez‐Olvera et al. 2024). Indeed, the only pathogenic lineage of tomato‐associated strains is *C. michiganensis* while the other nonpathogenic lineages are among the natural microbiota of tomato plants (Osdaghi, Taghavi, et al. 2018). All these taxonomic refinements raise the question of whether current detection methods are technically applicable to quarantine purposes and emphasise at the same time the need for re‐evaluation of those methods for sensitivity, specificity and reproducibility (EPPO 2016). This would help plant pathology agencies and tomato seed industry inspectors to specifically target the enemy and neglect the nonpathogenic lineages with lower cost and labour (see detection and identification section).

## 2 Disease Symptoms

**FIGURE 1:**
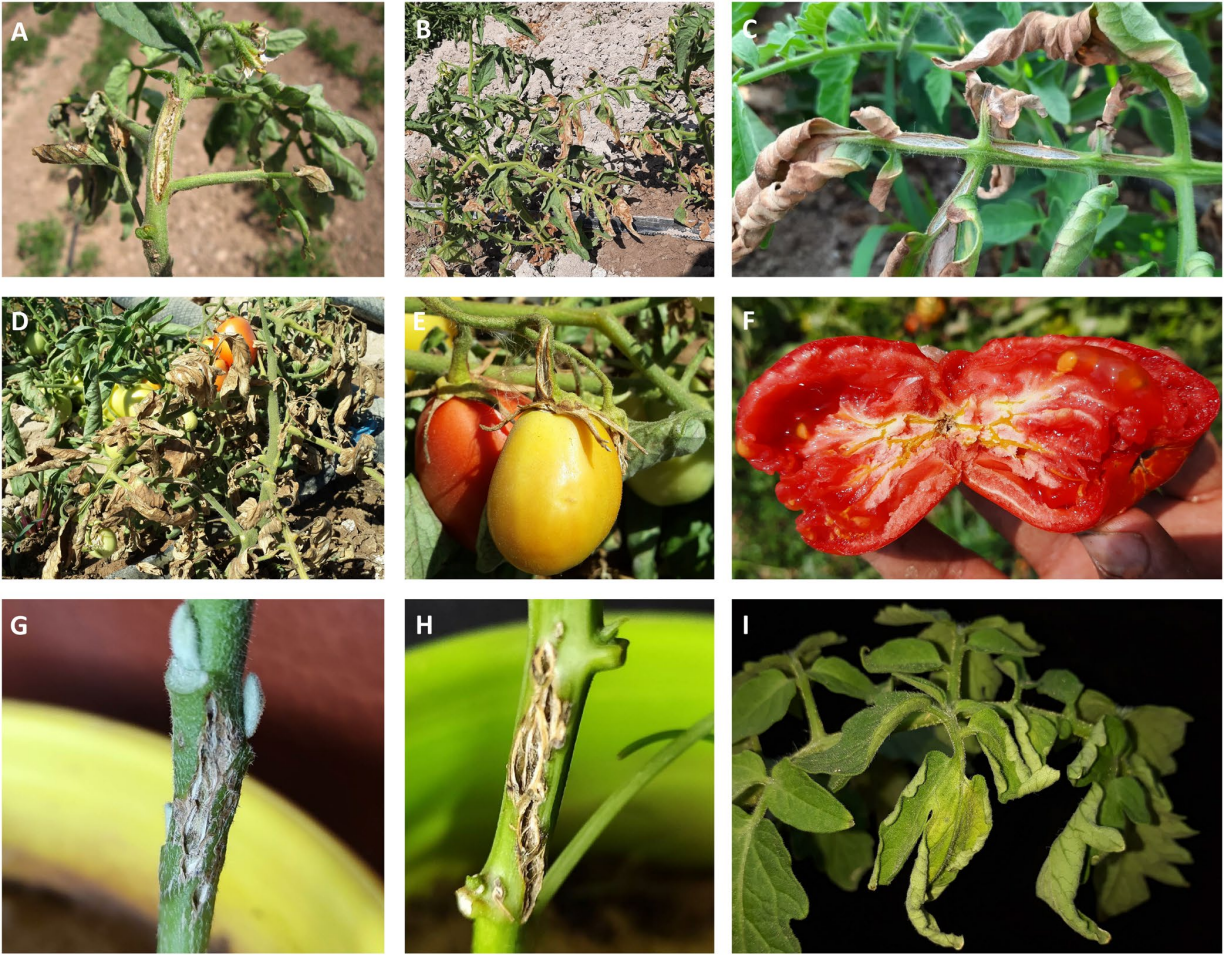
Field symptoms of tomato bacterial canker disease caused by *Clavibacter michiganensis* on tomato foliage (A–D) and fruits (E, F). Artificially inoculated eggplant and pepper plants show canker on the site of inoculation (G, H, respectively), while tomato plants show both stem canker and leaf wilting (I).

Symptoms of bacterial canker disease are diverse and are influenced by the pathway of infection, plant cultivar and age, as well as environmental factors, that is, temperature, humidity and nutrition (Gleason et al. 1993; Jones, Zitter, et al. 2014; Nandi et al. 2018). Infected seeds, infected tomato seedlings and transplants do not generally show any visible symptoms of the disease until maturity or unless they are exposed to high temperature, drought and physical stresses (Dhanvantari 1989; Gitaitis et al. 1991). Seedling symptoms start with small, white and raised spots on leaves. While infrequent, leaf margin discolouration and wilting may occur, leading to complete seedling wilt and plant death. Once transplanted in the field, the first symptoms on young plants include desiccation of the leaflet margin and overall plant wilt in severe cases of infections. Infected young seedlings become stunted and wither rapidly (Figure 1A–C). Numerous small whitish or tan pustules may appear on leaf veins, petioles and later on peduncles. However, the latter symptoms are observed only under high relative humidity (Jones, Zitter, et al. 2014).

Leaf symptoms are common in fully grown tomato plants, especially under warm and drought conditions. Leaf symptoms initially appear as interveinal, pale green, flaccid zones that quickly turn to yellow‐brown necrotic areas resembling sunburn (Figure 1B–D; Lamichhane et al. 2011). Marginal pale brown necrotic areas also appear on the leaves, with a scorched appearance. Leaf margins turn brown, with a yellow border, while lower leaves wilt, often only on one side. Veins on the leaves and petioles can become dark and sunken. Hydathodes of tomato leaves serve as extremely efficient infection entry points for epiphytic populations of the pathogen. Thus, chlorotic lesions progress at the tips of leaflet lobes about 2 weeks after inoculation of guttation droplets. Lesions expand along the leaflet margins and become necrotic (Carlton et al. 1998).

Characteristic symptoms of canker that lead to the name of the disease are observed on stems (Figure 1A). Light yellow to brown streaks or cankers appear on stems, becoming deeper and darker in cases of severe infections. As the disease progresses, infected stems split lengthwise and a pale yellow‐to‐reddish brown discolouration of the vascular tissue is observed (Figure 1A). Severely infected stems split, forming long brown cankers, while the upper parts of the branch begin to wilt and die. Brown streaks can be seen in the vascular system when the stem is cut open. Yellow sticky fluid may emerge from the cut stem when squeezed. In advanced infection, vascular discolouration is seen as brown streaks on the stem and petiole (Figure 1C). On cutting stems, petioles and peduncles, a creamy‐white yellow or reddish‐brown discolouration of vascular tissue and pith, and cavities within the pith will be evident. The pith of infected stems turns brown, granular to mealy and filled with cavities. Eventually, vascular wilting and premature death of entire plants are observed (Lamichhane et al. 2011). Symptoms on tomato fruits are rare, seen only under high relative humidity and cultivar susceptibility. Fruits present small (2–5 mm), creamy‐white spots with tan or brown centres called ‘bird eye spot’. Fruits may remain small and fall prematurely, or ripen unevenly. They also often show external marbling and internal bleaching of vascular and surrounding tissues (Figure 1F). Fruit symptom severity is in some cases unrelated to the severity of symptoms during vascular infection, suggesting different mechanisms for colonisation of different tissues (Peritore‐Galve et al. 2020, 2021).

Following artificial inoculations under greenhouse conditions (Figure 1G–I), the first symptom is a reversible wilting of leaves during hot weather, later becoming irreversible. The whole plant then desiccates. Leaves may show white interveinal areas, turning brown and necrotic generally before wilt symptoms appear (Haghverdi et al. 2025). In stem injection inoculation, the main symptoms are canker of the stem at the point of inoculation and the upward turning of one or a few of the leaves (Figure 1I). As the systemic infection progresses, the entire leaves may wilt and shrivel. Most *C. michiganensis* strains induce stem canker and wilting of more than one leaf or the entire plant, except for some strains that cause only canker (Malliarakis et al. 2023). Inoculation of eggplant and pepper under greenhouse conditions induces stem canker at the site of inoculation and is less likely to cause leaf symptoms and overall wilting (Figure 1G,H; Haghverdi et al. 2025). Disbudding and defoliation contribute to the secondary spread of bacterial canker in commercial greenhouses (Kawaguchi et al. 2010).

Although bacterial canker symptoms are fairly diagnostic depending on the environmental conditions, plant age and disease stage, wilt symptoms can be confused with several other diseases, that is, bacterial wilt caused by *Ralstonia solanacearum* , Fusarium wilt caused by *Fusarium oxysporum* f. sp. *lycopersici* and Verticillium wilt caused by *Verticillium dahliae*/*V. albo‐atrum* (Jones et al. 1991). Both bacterial canker and bacterial wilt diseases of tomato progress much more rapidly than fungal wilt diseases where the infected plant may be completely wilted within a few days of observing the initial symptoms (CABI 2021). *R. solanacearum* does not cause canker and cracks on the stems, nor is bird eye spot observed on the fruits. A quick and fairly reliable field diagnosis for bacterial wilt is to submerge a piece of stem (8–10 cm) containing vascular tissue in a clear glass jar containing clear water. If the plant has bacterial wilt, a white cloudy bacterial streaming can often be observed coming from the vascular tissue. On the other hand, bacterial wilt can cause a yellow to brown vascular discolouration, which is usually quite distinct from the darker red discolouration associated with Fusarium wilt. Symptoms of vascular discolouration with Verticillium wilt typically do not extend into the leaf petioles. Further, as symptoms progress, Verticillium wilt often causes a characteristic V‐shaped lesion on leaflets, with the V opening toward the leaflet margins. Necrotic leaflet lesions are often surrounded by a chlorotic margin (CABI 2021).

## 3 Host Range of the Pathogen

Nowadays, *C. michiganensis* is recognised as being a specialist plant pathogen presenting a very narrow host range. Tomato is the main host of *C. michiganensis* in both field‐ and greenhouse‐grown crops. However, recent findings showed that small variations in the genomic content of *C. michiganensis* strains drive host specificity of the bacterium (Verma et al. 2024). Natural infections by *C. michiganensis* have infrequently been reported on pepper ( *Capsicum annuum* ) as well as in other solanaceous species such as eggplant ( *Solanum melongena* ) and the wild nightshade species *Solanum douglasii* , *Solanum nigrum* and *Solanum triflorum* (Latin et al. 1995; Osdaghi 2020). Resistance in domesticated eggplant to *C. michiganensis* involves the recognition of a secreted putative serine hydrolase, ChpG (Verma and Teper 2022). Boyaci et al. (2021) noted that three cultivated eggplant genotypes were highly susceptible to the pathogen while 31 eggplant genotypes displayed no symptoms. *C. michiganensis* strains isolated from tomato showed reduced virulence on pepper and bell pepper compared to tomato plants. Symptoms on pepper and bell pepper due to artificial inoculation of tomato strains were limited to stem canker with no wilting symptoms (Yim et al. 2012; Haghverdi et al. 2025). It is difficult to recognise if the pepper infections reported before the reclassification of *C. michiganensis* sensu lato were due to the pepper pathogen *C. capsici* or by authentic strains of *C. michiganensis* . A number of solanaceous plants are reported to be susceptible upon artificial inoculation (Osdaghi 2020). *C. michiganensis* also induces disease symptoms on *Nicotiana benthamiana* (Hwang et al. 2024). Recently, Ignatov et al. (2019) reported the pathogenicity of *C. michiganensis* on potato ( *Solanum tuberosum* ) under natural conditions in Russia. The *C. michiganensis* strains isolated from potato were distinct from the potato pathogen *C. sepedonicus* and were pathogenic on both tomato and potato under greenhouse conditions (Ignatov et al. 2019; Osdaghi et al. 2022).

## 4 Economic Impact of the Disease

According to the EPPO Global Database https://gd.eppo.int/taxon/CORBMI/distribution and CABI Compendium https://www.cabidigitallibrary.org/doi/10.1079/cabicompendium.15338, bacterial canker of tomato is widespread worldwide, except for Antarctica (Osdaghi 2020). Bacterial canker, along with bacterial spot (caused by four *Xanthomonas* groups) and bacterial wilt (caused by *R. solanacearum* ) are the most important bacterial diseases of tomatoes (Osdaghi et al. 2017, 2021). Yield losses due to bacterial canker infection may reach 93% plant death. Up to 50% average fruit weight decreases have been reported in the literature due to bacterial canker infection (Chang et al. 1992b). Hausbeck et al. (2000) estimated losses of up to $300,000 in Michigan (United States) from sporadic epidemics of the disease. Yield losses and the economic impact of bacterial canker disease are correlated with plant age and phenology when infection occurs. According to Chang et al. (1992b), yield and average fruit weight of processing tomatoes (cv. Heinz1810) were related to the incidence of systemic infection 1 week before harvest. In plants infected during clipping or seedling harvest, yield decreased by 46% and average fruit weight decreased by 13 g when the highest incidence of systemic infection was 31%–83%. Thus, a 5%–7% yield decrease was estimated for each 10% increase in bacterial canker incidence (Chang et al. 1992b). Plant survival and yield in the field are severely affected when transplants have a pathogen population of > 10^8^ CFU/g of tissues. Under artificially inoculated field conditions, inoculated plants produced yields that were 51%–63% of those produced by uninoculated controls (Hausbeck et al. 2000). It has also been noted that the yield of infected plants was compensated by adjacent healthy plants (Ricker and Riedel 1993). Bacterial canker infection seems to affect fruit ripening as well. When the highest incidence of systemic infection was 31%–83%, the percentage of green fruits decreased by 41% and the percentage of ripe fruits increased by 41% (Chang et al. 1992b).

## 5 Bacteriological Features of the Pathogen

**FIGURE 2:**
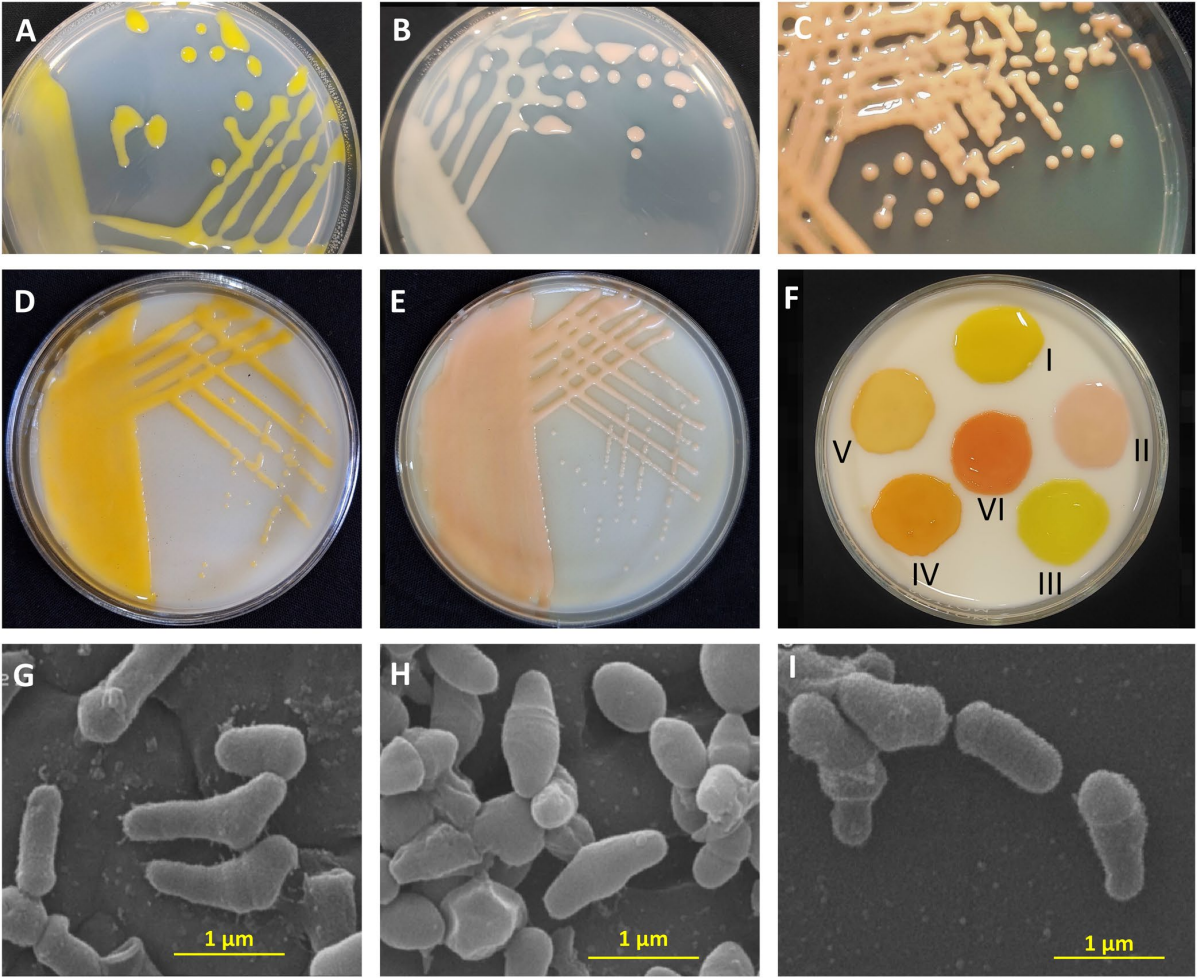
Bacterial canker pathogen differs from the other members of *Clavibacter* in colony pigmentation. Colonies of yellow‐ (A) and pink‐pigmented (B) strains of *Clavibacter michiganensis* on YPGA (A, B) and YDC (D, E) media compared to the tomato‐nonpathogenic strains of *Clavibacter lycopersici* (C and F). Bacterial cultures in section ‘F’ include yellow‐ and pink‐pigmented *C. michiganensis* (I, II), *Clavibacter tessellarius* (III), *Rathayibacter* sp. (IV), *Clavibacter lycopersici* (V) and *Clavibacter zhangzhiyongii* (VI). Scanning electron microscopy (SEM) of yellow‐ (G: ICMP 2550^T^) and pink‐pigmented (H: CFBP 9078) *C. michiganensis* as well as peach‐pigmented *C. lycopersici* (I: CFBP 8615^T^) strains showed curved and club‐shaped rod cells. Yellow bars (1 μm) show image scale in G, H and I.

Plant‐pathogenic coryneform bacteria are well known for producing a variety of lipid‐soluble carotenoid pigments on culture media (Schaad et al. 2001; Hamidizade et al. 2020; Osdaghi, Young, et al. 2020, Osdaghi et al. 2024). General characteristics of *C. michiganensis* are similar to those of other *Clavibacter* species possessing gram‐positive, curved rod (coryneform), non‐spore‐forming cells (Davis et al. 1984). The cells are negative for anaerobic growth, levan production, pectinolytic activity on potato slices and hydrolysis of Tween 80. *C. michiganensis* is known to produce domed round shiny mucoid colonies on general and semiselective culture media, for example, yeast extract‐peptone‐glucose agar (YPGA), yeast extract‐dextrose‐calcium carbonate (YDC) agar (Figure 2A–F) and bacterial canker of tomato (BCT) (Schaad et al. 2001; Ftayeh et al. 2011). Before the genomics era, it was thought that the bacterial canker pathogen included phenotypically diverse strains with different colony pigmentation. For instance, Kaneshiro et al. (2006) noted that atypical tomato‐associated *Clavibacter* strains produced white colonies with mucoid consistency, pink and mucoid colonies, yellow and less fluid colonies than typical (dry or sticky) and orange and sticky colonies. In some cases, only colony morphology but not pigmentation was stated in the literature, where Waleron et al. (2011) reported that the colonies of all tested *C. michiganensis* strains were mucoid.

Before the comprehensive reclassification of *C. michiganensis* sensu lato, morphological variants of *C. michiganensis* have frequently been reported in the literature, though the correlation between colony characteristics and pathogenicity on tomato was not clarified. According to Zaluga, Van Vaerenbergh, et al. (2013) *Clavibacter* strains originating from tomato in Belgium showed differences in colony morphology. Most of the *C. michiganensis* sensu lato strains studied previously displayed typically yellow fluid colonies, while some atypical strains, for example, LMG 26808 and LMG 26809, showed more orange fluid colonies. Furthermore, strains isolated from pepper (nowadays belonging to *C. capsici*) in Korea produced orange‐pigmented colonies with lower mucoidy than typical *C. michiganensis* strains isolated from tomato (Yim et al. 2012). Another set of tomato‐associated *Clavibacter* strains with yellow‐orange and peach‐pigmented round mucoid colonies that have traditionally been referred to as *C. michiganensis* were recently reclassified as *C. californiensis* , *C. phaseoli* and *C. lycopersici* (Yasuhara‐Bell and Alvarez 2015; Arizala et al. 2022). Thus, the current authentic *C. michiganensis* includes mostly yellow‐pigmented colonies on general culture media. Accordingly, diagnostic guidelines issued by EPPO, International Seed Testing Association (ISTA) and European Food Safety Authority (EFSA) refer to the bacterial canker pathogen as a solely yellow‐pigmented bacterium (Figure 2A,D; EFSA 2014; EPPO 2016). However, a pink‐pigmented tomato‐pathogenic variant of the pathogen has recently been isolated from tomato seeds and leaves in southern Iran, expanding the phenotypic range of the pathogen (Figure 2B,E; Haghverdi et al. 2025). This raises the need for reconsideration in diagnostic guidelines and detection procedures to ensure precise screening of tomato seeds and plant materials in quarantine ports (see Section 11). Despite their differences in colony morphology and pigmentation, scanning electron microscopy (SEM) imagery of tomato‐associated *Clavibacter* strains with different colony pigmentation indicated similar cell morphology and dimensions. All tomato‐associated *Clavibacter* lineages possess curved and club‐shaped rod cells, occasionally being arranged at an angle to give a V formation (Figure 2G–I).

## 6 Biology of the Pathogen

### 6.1 Inoculum Sources

**FIGURE 3:**
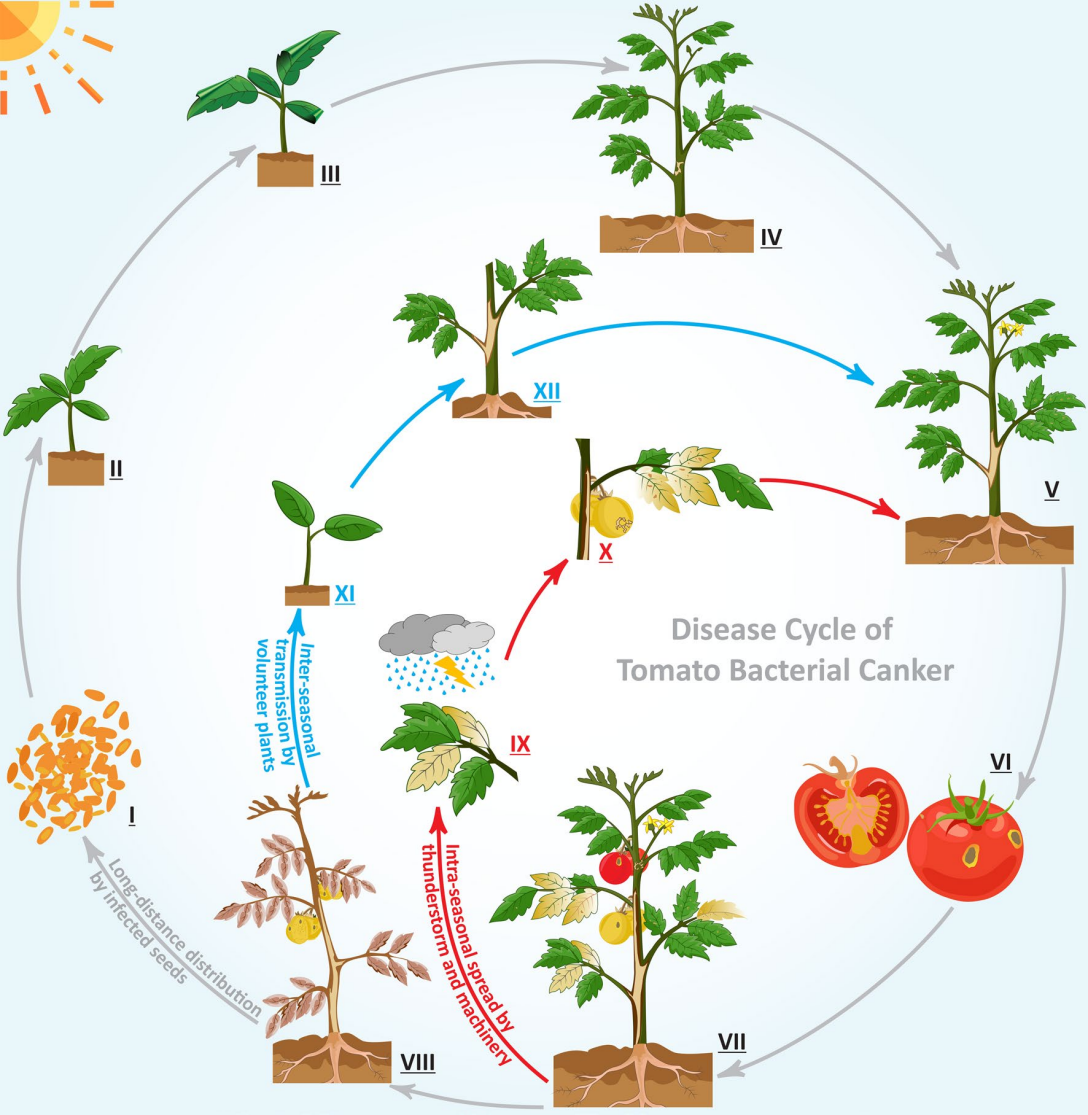
Disease cycle of tomato bacterial canker caused by *Clavibacter michiganensis* under natural conditions. Long distance dissemination of the pathogen occurs via movement of latently infected tomato seeds and transplants (primary infection, I–VIII), while contaminated machinery, rainstorms and plant handling initiate short distance spread of the pathogen (secondary infection, IX and X). Interseasonal transmission of the pathogen is driven by volunteer tomato plants and alternative hosts (XI and XII).

*Clavibacter michiganensis* is a seedborne pathogen capable of being introduced into areas with no history of the disease via infected seed lots (Anwar et al. 2004). Low levels of seed lot infection (as low as 1 in 10,000 seeds) may lead to epidemic development as cultural practices can cause secondary dissemination of the pathogen (Chang et al. 1991) (Figure 3I–VIII: primary infection). Very low bacterial population size, as low as 5 CFU per seed, can give rise to infection of seedlings (Lelis et al. 2014). It has been noted that weakly virulent and avirulent *C. michiganensis* strains are also frequently isolated from seeds and plants. The bacterial canker disease is transmitted from infected seeds to seedlings and mechanically from plant to plant during seedling production, grafting, pruning and harvesting (Xu et al. 2010).

Infected transplants, tomato debris, alternative hosts and tomato volunteers may also serve as primary or secondary inoculum sources (Chang et al. 1992a) (Figure 3IX–XII). Variations in time and routes of infection affect the range of symptoms under natural conditions. In the case of primary infections where plants are infected at early stages of their life, the disease becomes systemic, affecting fruit quality and yield, which typically leads to plant death. Secondary infection, however, includes only foliar symptoms, such as chlorosis of leaves and may affect the quality and yield of the current crop (Nandi et al. 2018). Infected host debris, including roots, might be an important inoculum source of the pathogen for the next season in greenhouses. However, under natural field conditions, the most likely sources of primary inoculum are nightshade hosts (Moffett and Wood 1984).

### 6.2 Pathogen Survival

Survival time of the pathogen in crop residues under field conditions is variable, ranging from 2 months in Morocco to 2 years in Iowa, United States (Fatmi and Schaad 2002). In host debris left on the soil surface, bacteria survived 120–260 days for crop production cycles that ended in winter and 45–75 days for those that ended in summer. In stems or roots buried in winter, this period was 45–75 days (Vega and Romero 2016). Soil temperature has a greater effect on survival than moisture. When the stem pieces were buried in soil kept at constant temperature (22°C) and moisture, survival was longer but still limited to 11 months (Moffett and Wood 1984). Higher temperature treatment (45°C) can cause a significant reduction in the pathogen population even after 1 week (Zanón and Jordá 2008). Moffett and Wood (1984) noted that under a crop rotation strategy, it is extremely unlikely that inoculum for outbreaks of the disease will come from buried crop debris. Jacobs et al. (2005) evaluated the role of pigmentation in population dynamics, leaf colonisation strategies and field survival of *C. michiganensis* . Pigment‐deficient *C. michiganensis* mutants were significantly reduced in UVA (320–400 nm) radiation survival in vitro and showed reduced field populations on peanut when compared to the wild‐type strain.

### 6.3 Viable but Non‐Culturable State of the Pathogen

In response to adverse environmental conditions, *C. michiganensis* may enter the viable but non‐culturable (VBNC) state that allows persistence in unfavourable conditions and resuscitation once the stress is diminished (Jiang et al. 2016). The VBNC state limits pathogen detection by culture‐based assays and subsequent resuscitation of *C. michiganensis* cells in tomato seedlings yet poses a risk for disease development in the field (Chen, Bai, et al. 2021). Jiang et al. (2016) noted that CuSO_4_ in oligotrophic conditions and low pH induces the VBNC state in *C. michiganensis* , where some of the VBNC cells retained their ability to colonise tomato seedlings but failed to produce typical bacterial canker symptoms by 2 months post‐inoculation. Rel, an enzyme that mediates the synthesis of the secondary messenger guanosine tetraphosphate/pentaphosphate (ppGpp), accumulates after exposure of *C. michiganensis* cells to stressful conditions, leading to cells entering the VBNC state, which can be seen as a way of reallocating cellular resources to counter the stressful conditions (Bai et al. 2022).

## 7 Disease Cycle of Tomato Bacterial Canker

Pathogen penetration occurs mainly through trichomes and hydathodes (Chalupowicz et al. 2017). Physical wounds during seedling production and crop maintenance facilitate the pathogen entry (Xu et al. 2012). Tomato flower infection may lead to fruit infection. Seed infection occurs from xylem colonisation, which can originate from fruit lesions (Tancos et al. 2013). For primary infection of the seedlings, the ‘window of vulnerability’ ranges from transplanting to the 17‐ to 18‐leaf stage (Figure 3II,III). Plants inoculated after the vulnerability period express disease symptoms but do not wilt or die (Sharabani, Manulis‐Sasson, et al. 2013; Sharabani, Shtienberg, et al. 2013). The approximate incubation period of symptomless infected tomato seedlings is 10 days (Kawaguchi et al. 2023). Maximum disease incidence on fruits under greenhouse conditions resulted when 10^8^ CFU/mL of inoculum was sprayed over flowers twice and 3 days apart. The probability of infection of healthy tomato plants is 75% after cutting with scissors soaked in a cell suspension of 10^6^ CFU/mL (Kawaguchi et al. 2022). In stem base drop‐inoculated plants, the pathogen was found to be present in low densities in roots, stems and leaves only 3 h after inoculation (Lelis et al. 2014). Bacteria multiplied rapidly in cotyledon petioles that remained after clip inoculation and they moved in the stem toward both roots and shoots, leading to decreased root development (Xu et al. 2012). A bioassay using a green fluorescent protein‐labelled strain showed that the bacteria extensively colonise the lumen of xylem vessels and preferentially attach to spiral secondary wall thickening of the protoxylem (Chalupowicz et al. 2012).

Movement of *C. michiganensis* from the inoculated leaflet into the rachis is slow and erratic. Upon entrance, the bacteria first multiply within intercellular spaces lying beneath the stomata (Carlton et al. 1998). In grafted seedlings when either rootstock or scion is infected via a contaminated grafting knife, bacteria are translocated in both directions from the graft union at high inoculum doses (Xu et al. 2010). While the hydraulic radius of xylem vessels is not affected, the stem‐specific and the leaf‐specific conductivity are significantly reduced by inoculation. The pathogen reduces the growth and alters plant‐water relations of tomato plants by reducing the stem hydraulic conductivity, as a consequence of the formation of biofilms that restrict xylem sap flow (Romero et al. 2018). Acropetal movement of the pathogen resulted in extensive systemic colonisation of the whole plant reaching the apical region after 15 days (Chalupowicz et al. 2012).

According to Huang and Tu (2001), when tomato plants in hydroponic culture were inoculated with the pathogen through wounds on the stems, the bacteria moved downward from the inoculation site to the roots and were released from the roots into the nutrient solution. Infections by epiphytic *C. michiganensis* populations can also occur under a wide range of temperatures, wetness and seedling age (Frenkel et al. 2016). Exudation through guttation leads to the formation of epiphytic populations on leaflets. The pathogen is exuded in large numbers in the guttation fluid of infected plants (Sharabani, Manulis‐Sasson, et al. 2013; Sharabani, Shtienberg, et al. 2013). The temperature during the initial stages of *C. michiganensis* infection affects bacterial canker development and virulence gene expression. A highly significant correlation was found between the average temperatures during the first month after inoculation and the time taken for 50% of the plants to wilt or die (T50), where the shortest T50 mortality (70 days) was observed for an average temperature of 26°C (Sharabani et al. 2014).

Real‐time investigation of germinating seeds revealed that *C. michiganensis* aggregates on hypocotyls and cotyledons at an early stage of germination (Xu et al. 2010). The approximate concentration of *C. michiganensis* in symptomless infected plants was determined as 3 × 10^6^ CFU/g plant tissue (Kawaguchi et al. 2022). Dutta et al. (2014) noted that the seeds of non‐host plants can become infested with incompatible and null‐interacting bacterial species through flower colonisation, and they can be transmitted via epiphytic colonisation of seedlings. It has been noted that weakly virulent and avirulent *C. michiganensis* strains are also frequently isolated from seed and plants. For instance, Alvarez et al. (2004) reported that 81% of the *C. michiganensis* sensu lato strains isolated from tomato seed were hypovirulent or avirulent. However, most of these strains were later reclassified as novel stand‐alone species (Yasuhara‐Bell and Alvarez 2015).

## 8 Genetic Diversity and Population Structure

Various DNA fingerprinting methods have been used to reveal *C. michiganensis* population structure. The repetitive sequence‐based PCR (rep‐PCR) method was used in the 1990s to distinguish strains of the tomato pathogen. At least four types (A, B, C and D) were differentiated within *C. michiganensis* , and it was observed that this technique was unable to differentiate pathogenic and nonpathogenic tomato‐associated *C. michiganensis* strains (Louws et al. 1998). No relationship was found between rep‐PCR clustering and the year/location of strains (Wassermann et al. 2017). Based on polyphasic characterisation and phylogenetic analysis, Jacques et al. (2012) showed that *C. michiganensis* is monophyletic and is distinct from its closest taxonomic neighbours. Evolutionary genome analysis provided evidence that the tomato bacterial canker pathogen emerged after a host shift from grasses. Comparative genomics and phylogenomics analyses identified conserved loci that make *C. michiganensis* a successful pathogen during the transition between these hosts (Osdaghi, Rahimi, et al. 2020; Yañez‐Olvera et al. 2024). They also noted that tomato‐associated nonpathogenic *Clavibacter* strains were phylogenetically distinct from the pathogenic strains while cross‐reacting with *C. michiganensis* identification tools. Multilocus sequence analysis and typing (MLSA/MLST) showed that *C. michiganensis* clonal complexes linked pathogenic strains from highly diverse geographical origins and strains isolated over long periods of time in the same location (Jacques et al. 2012). MLST of *C. michiganensis* strains isolated in Uruguay using the same MLST scheme as the study of Jacques et al. (2012) revealed novel sequence types in this country that could reflect the introduction of new strains from different origins, most likely from seed importation (Croce et al. 2016). Similar analyses divided a worldwide collection of 184 *C. michiganensis* strains into two phylogroups I and II (Ansari et al. 2019). Phylogroup I clustered all strains isolated in eastern Asia (Taiwan and China), eastern Europe (Hungary and Slovenia), South Africa and Portugal, while the strains isolated in Brazil, Italy and Spain were clustered in phylogroup II (Ansari et al. 2019). Multilocus variable‐number‐tandem‐repeats (VNTR) analysis (MLVA) developed by Zaluga, Stragier, et al. (2013) distinguished 25 haplotypes within *C. michiganensis* . Based on MLSA and MLVA, *C. michiganensis* strains from central Chile were found to exhibit low genetic diversity, and sequence types match strains from other parts of the world (Valenzuela et al. 2018). Baysal et al. (2011) introduced intersimple sequence repeats (ISSR) primers to characterise *C. michiganensis* .

## 9 Genomic Features

**FIGURE 4:**
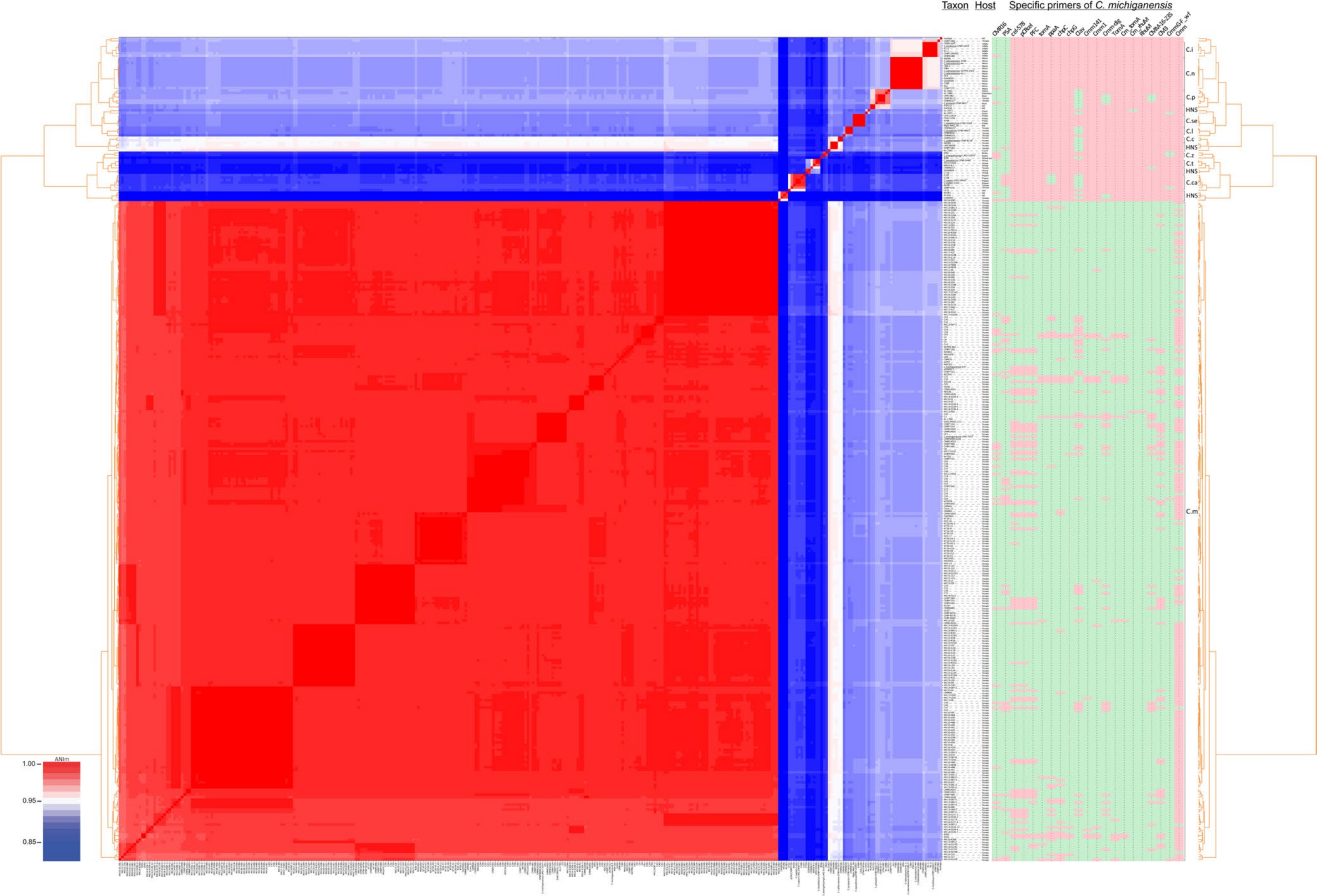
Phylotaxonomic analysis of all publicly available genome resources of *Clavibacter* spp. using pyANI v0.2.11 Python pipeline. All tomato‐pathogenic strains of *Clavibacter michiganensis* clustered in a monophyletic clade (C.m) while nonpathogenic tomato‐associated strains were scattered among the other *Clavibacter* lineages. Results of in silico PCR using the sequence of PCR primers for *C. michiganensis* (right side of the figure) showed that the primer pairs Cmm141F/Cmm141R, Cmm1F/Cmm1R, RhuM‐F/RhuM‐R, CM3/CM4, cel‐578up/cel‐2752low, PFC3/PFC5, pCRcel‐593/pCRcel‐1860, tomA‐F/tomA‐R, Cm_tomA_F/Cm_tomA_R, TomA‐F1/TomA‐F2, chpC‐F/chpC‐R and chpG‐F/chpG‐R are highly specific for *C. michiganensis* . Phylogenetic clades indicated by abbreviations include C.i: *C. insidiosus* , C.n: *C. nebraskensis* , C.p: *C. phaseoli* , C.se: *C. sepedonicus*, C.l: *C. lycopersici*, C.c: *C. californiensis* , C.z: *C. zhangzhiyongii*, C.t: *C. tessellarius*, C.ca: *C. capsici* and C.m: *C. michiganensis* . The clades designated as HNS are hypothetical new species lacking valid taxonomic description (Osdaghi, Rahimi, et al. 2020).

*Clavibacter michiganensis* is the most studied and richest corynebacterial plant pathogen in terms of available genomic resources. By April 2024, 333 complete or draft genome sequences designated as *Clavibacter* had been deposited in the NCBI GenBank database. Preliminary screening showed that among the 333 genomes, 327 strains were authentically *Clavibacter* and 282 strains were isolated from tomato. Average nucleotide identity (ANI)‐based calculations assigned 265 strains into *C. michiganensis* , all originating from tomato plants/seeds (Figure 4). The remaining 17 tomato‐associated *Clavibacter* strains were scattered among other species, in some cases standing alone as a hypothetical new species. For instance, the following clades include tomato‐associated strains: *C. insidiosus* (CFBP 6488), *C. phaseoli* (CFBP 8217 as well as all members of the taxon originally described as *C. michiganensis* subsp. *chilensis*; Yasuhara‐Bell and Alvarez 2015), *C. californiensis* (CFBP 8216^T^, A6099, CFBP 7493), *C. lycopersici* (CFBP 8615^T^ and CFBP 8616), *C. capsici* (RA1B). The strains CASJ009 and MX14‐G9D belong to a hypothetical new species (Figure 4, Osdaghi, Rahimi, et al. 2020). The bacterial canker pathogen entered the genomics era when the complete genome sequence of the reference *C. michiganensis* strain NCPPB 382 became available (GenBank: AM711867.1; Gartemann et al. 2008). Strain NCPPB 382 has a 3298 Mb circular chromosome with high G + C content (72.6%) and 3080 putative protein‐encoding sequences (CDSs), similar to those of other corynebacterial plant pathogens (Chen, Khojasteh, et al. 2021). The complete genome sequence of the type strain of *C. michiganensis* LMG 7333^T^ is also available under the accession number NZ_MZMP01000000 (Oh et al. 2022).

## 10 Pathogenicity Mechanisms

Before the establishment of genome‐based studies, extracellular polysaccharide (EPS) production has been underlined as a determinative pathogenicity feature of *C. michiganensis* . Van den Bulk et al. (1990) noted inhibition of callus development from protoplasts of *Solanum peruvianum* by EPS of *C. michiganensis* . EPS‐producing strains efficiently colonised tomato plants and induced a hypersensitive reaction (HR) when injected into leaves of the non‐host *Mirabilis jalapa* (four o'clock plant). In contrast, the strains producing only small amounts of EPS in vitro were avirulent, did not propagate in tomato, and failed to induce an HR on *M. jalapa* (Eichenlaub et al. 1991). Hibberd et al. (1996) demonstrated that the ability to induce an HR in *M. jalapa* is under the control of a single dominant gene. The HR‐inducing activity present in *C. michiganensis* cell supernatant is heat stable, sensitive to proteases and has an apparent molecular mass in the range of 35 to 50 kDa (Alarcon et al. 1998). The HR‐inducing factor in *M. jalapa* was identified as the serine protease ChpG (Stork et al. 2008). While *C. michiganensis* grows mainly in the xylem of tomato plants, no significant reduction was found in genome size and CDSs compared to soil‐inhabiting coryneform bacteria (Gartemann et al. 2008). Comparative analysis revealed that tomato seedborne nonpathogenic strains do not possess plasmids pCM1 and pCM2. Also, they lack the majority of important virulence factors described so far for pathogenic *C. michiganensis* , such as the 129‐kb low G + C content region known as a pathogenicity island, harbouring *chp*/*tomA,* near the chromosomal origin of replication, which is necessary for successful colonisation and pathogenicity on tomato (Załuga et al. 2014; Osdaghi, Rahimi, et al. 2020). Gartemann et al. (2008) and Chalupowicz et al. (2010) identified mutant clones of the pathogenic strain NCPPB 382 that lack the entire *chp*/*tomA* pathogenicity island (CMM30‐18 and Cmm27), rendering them completely nonpathogenic. Meletzus et al. (1993) noted that the strain NCPPB 382 carries two plasmids (pCM1 of 27.35 kb and pCM2 of 72 kb) carrying genes involved in virulence. Similar to the 129‐kb chromosomal pathogenicity island, the two circular plasmids have lower G + C contents, with pCM1 of 66.5% and pCM2 of 67.6% (Gartemann et al. 2008). After curing the two plasmids, derivatives exhibited a reduced virulence but were still proficient in the ability to colonise the host plant and in the production of EPS (Meletzus et al. 1993). Chalupowicz et al. (2017) showed that plasmid‐borne genes, which have a crucial role in wilting, are not required for blister formation. When one of the two plasmids is lost, there is a significant delay in the development of wilting symptoms after infection. The plasmid‐borne virulence genes are *celA* on pCM1 and *pat*‐*1* on pCM2. The gene *celA* plays a major role in pathogenicity as it encodes an endo‐β‐1,4‐glucanase, CelA (Jahr et al. 2000), while strains with or without *pat*‐*1* (pCM2) are indistinguishable based on disease severity (Bella et al. 2012; Thapa et al. 2017). Burger et al. (2005) demonstrated the presence of *pat*‐*1* homologous nucleotide sequences on the chromosome and on plasmid pCM2. The introduction of the *pat*‐*1* region into an endophytic plasmid‐free *Clavibacter* strain converted the latter strain into a virulent strain (Dreier et al. 1997). Burger et al. (2005) identified the serine protease Pat‐1 in *C. michiganensis* , which led to the finding of a family of serine proteases (ChpA–ChpG) that play a role in colonisation of tomato (Stork et al. 2008). In *C. michiganensis* NCPPB 382, the genes *chpA*, *chpB* and *chpD* are pseudogenes containing frameshifts and/or in‐frame stop codons. In planta, the titre of the *chpC* mutant was drastically reduced and only weak disease symptoms were observed. However, the *chpG* mutant was not impaired in virulence. *chpC* was the first chromosomal gene with a defined role in plant– *C. michiganensis* interaction (Stork et al. 2008). Hwang et al. (2019) also found that the cellulase catalytic domain and cellulose‐binding domain of CelA together were sufficient for both cellulase activity and full virulence of the bacterium. Interestingly, a *celA* orthologue from *C. sepedonicus* can restore the full virulence to the *celA* mutant of *C. michiganensis* (Hwang et al. 2019). The *celA* and *pat*‐*1* genes were not detected in pepper‐pathogenic strains recently renamed as *C. capsici* (Yim et al. 2012; Valenzuela et al. 2021). Transcriptional analysis revealed that *celA* and *pat*‐*1* are significantly induced 12–72 h post‐inoculation (hpi), whereas *chpC* and *ppaA* are highly expressed only 96 hpi. Transcription of chromosomal genes involved in cell wall degradation (*pelA1*, *celB*, *xysA* and *xysB*) is also induced at early stages of infection (Chalupowicz et al. 2010).

In subsequent years, additional genes have been identified as having a significant role in the *C. michiganensis* pathogenicity, for example, transcriptional regulators Vatr1 and Vatr2 (Savidor et al. 2012). According to Savidor et al. (2014), Vatr1 and Vatr2 regulate the expression of virulence factors, membrane and secreted proteins and signal‐transducing proteins. It has been noted that Pat‐1 is an immune elicitor inducing HR in *Nicotiana tabacum* (non‐host), but in tomato it acts as a virulence factor (Dreier et al. 1997; Hwang et al. 2022). The bacterium expresses Pat‐1 and CelA not only after host cell contact in planta but also in M9 minimal and xylem‐mimicking medium (Hiery et al. 2015). Recently, Hwang et al. (2024) found that the chromosomal *cviA1* gene in *C. michiganensis* plays an important role in necrosis development in *Nicotiana benthamiana* leaves. *cviA1* encodes a 180‐amino acid protein with a signal peptide at the N‐terminus and two putative transmembrane domains at the C‐terminus. Deletion of the signal peptide or the C‐terminus, including the two putative transmembrane domains in CviA1, failed to restore full necrosis in the mutant. Verma et al. (2024) demonstrated that the ChpG^C^ variant is not recognised as an immune elicitor in eggplant, whereas other ChpG variants from eggplant‐nonpathogenic strains are. They identified five *chpG* allelic variants within *C. michiganensis* populations and named them as *chpG* ^A^, *chpG* ^B1^, *chpG* ^B2^, *chpG* ^C^, and *chpG* ^D^. The *chpG* ^C^ variant was only present in the eggplant‐pathogenic strains of the pathogen. A single amino acid substitution in the ChpG serine protease domain eliminates its recognition in eggplant. *chpG* ^C^ has a single T506G substitution that results in a V169G amino acid alteration. The *chpG* ^C^ also elicits attenuated HR in the non‐host plant *M. jalapa*.

The availability of microarray, total RNA‐sequencing (RNA‐seq) and CRISPR/Cas9‐mediated gene‐editing techniques further clarified the gene expression profile of *C. michiganensis* during tomato infection. Flügel et al. (2012) constructed and validated a *C. michiganensis* oligonucleotide microarray and found that among 9254 tomato genes represented on the array, 122 were differentially expressed in infected plants compared to non‐inoculated plants. RNA‐sequencing indicated that numerous genes involved in stringent response, copper resistance and stress resistance were up‐regulated, and some involved in cell division were down‐regulated significantly (Bai et al. 2022).

Like Chen et al. (2022), Stevens et al. (2021) developed a suite of tools for genetic manipulation in *C. michiganensis* , including the *codA*::*upp* counterselection system to create markerless deletions in *Clavibacter*, an integrative plasmid and an R package for the identification of permissive sites for plasmid integration. The vector pSelAct‐KO is a recombination‐based marker‐less knockout system that uses dual selection to engineer seamless deletions of a region of interest, providing opportunities for repeated higher‐order genetic knockouts. Chen et al. (2022) detailed a highly efficient unmarked CRISPR/Cas9‐mediated gene‐editing system in *Clavibacter* that couples the expression of *cas9* and single‐guide RNA with homology‐directed repair templates and the negative selectable marker *codA*::*upp* within a single plasmid.

## 11 Detection and Identification of the Pathogen

Historically, detection of *C. michiganensis* in tomato seeds and plant specimens was based on culture plating on general or semiselective media. Vidaver (1982) and Gleason et al. (1993) summarised all conventional methods for detection and diagnosis of *C. michiganensis* in the pregenomics era, which mostly included culture‐based isolation and immunodiagnostic techniques. Since the early 1990s, DNA sequence‐based detection methods such as specific PCR, nucleotide probes and quantitative real‐time PCR have been extensively used for detection of the pathogen with higher efficiency and lower effort. The French National Laboratory for Plant Health initiated an international effort on the assessment of the methods used in laboratories for detection of *C. michiganensis* in tomato seeds, that is, dilution plating on semiselective media and immunofluorescence, including an interlaboratory study on naturally and artificially contaminated seeds by eight laboratories from six European countries (Olivier et al. 2010). A comparison of different methods was performed with reference strains to determine the applicability of the molecular tests in optimal conditions (Jacques et al. 2012). These comprehensive assays led to the preparation of the EPPO, ISTA and EFSA diagnostic guidelines (EFSA 2014; EPPO 2016).

### 11.1 Seed Extraction

Infected seed is often considered to be the primary inoculum source and the major source for outbreaks of bacterial canker. Owing to the fact that population levels of the pathogen in/on the seed may be very low, development of an efficient, sensitive and reliable seed extraction procedure is a prerequisite for the establishment of robust quarantine programmes. Seed extraction methods that include grinding the seeds are better at detecting the pathogen than methods that use only soaking (Hadas et al. 2005). This could be due in part to the fact that grinding exposes the interior parts of the seeds to culture media or solution buffer. Seedlots infested with fewer than 58 CFU/g do not cause disease under greenhouse conditions, whereas seeds with 1000 CFU/g caused disease in 78 out of 2000 test plants (Hadas et al. 2005). For a successful seed extraction, 24 g of the seed sample (approximately 10,000 seeds) is placed in a sterile double‐layered plastic bag containing 150 mL sterile phosphate‐Tween buffer. The specimen is incubated at 4°C for 15 min. Then the bag is placed with its contents in a stomacher (Lab Blender Model 400 Mark II) and blended for 15 min. The resulting suspension can be streaked onto semiselective culture media or directly used for PCR assays (Osdaghi 2020).

### 11.2 Culture‐Based Methods

Bacterial canker of tomato can be diagnosed by the field symptoms and by isolation of the causal organism on a nonselective medium or a semiselective medium followed by a pathogenicity test on a 2‐ to 4‐leaf‐stage tomato seedling (EPPO 2016). The most suitable part of tomato plants for reliable detection of the bacterium is the lower stem region (Krämer and Griesbach 1995). Semiselective media are useful for detection and isolation of *C. michiganensis* in plant health surveys and quarantine inspections. Within the past three decades, several semiselective culture media have been developed mainly on the basis of original medium NBY. The EPPO diagnostic guideline (2016) provided a detailed list of semiselective media and their ingredients for isolation of *C. michiganensis* . Modified CNS agar is a semiselective medium on which *C. michiganensis* colonies appear in 6–7 days (Vidaver and Davis 1988), while on SCM agar grey‐to‐black speckled colonies are formed (Fatmi and Schaad 1988). A modification of SCM agar, m‐SCM agar, yields clear colonies with yellow flecks that appear 7–9 days post‐incubation (Waters and Bolkan 1992). Ftayeh et al. (2011) noted that all previously published semiselective media (D2, KBT, D2ANX, SCM, mSCM, CMM1 and mCNS) gave false‐negative results. Thus, they developed a new selective and highly sensitive medium for isolation of *C. michiganensis* from seeds and latently infected plants and named the new medium BCT (bacterial canker of tomato). Exclusively, BCT also supports growth of the closely related species *C. insidiosus* , *C. nebraskensis* and *C. tessellarius* (Osdaghi, Robertson, et al. 2023). On CMM1 agar, colonies of *C. michiganensis* are yellow, mucoid and convex, while on BCT agar typical colonies appear creamy to yellow in colour, convex and shining (Kaneshiro et al. 2006; Ftayeh et al. 2011). A xylem‐mimicking medium (XMM) was also developed by Hiery et al. (2013) based on an apoplast medium for tomato‐pathogenic xanthomonads. In contrast to the apoplast medium, XMM contains no sugars but amino acids that serve as a nitrogen and carbon source. Altogether, the BCT medium seems to be more suitable for isolation of the bacterial canker pathogen despite its complex ingredients and application of rare antibiotics.

Kaneshiro et al. (2006) noted that a large proportion of *Clavibacter* strains associated with naturally infested tomato seeds were putatively hypovirulent or nonvirulent. Upon isolation of the suspected bacterial strains, pathogenicity tests should be carried out on tomato seedlings at 24**°**C–27°C under 60%–80% relative humidity. Inoculation techniques include infiltrating a freshly prepared aqueous suspension of bacterial cells (10^6^ CFU/mL) with a syringe into the stem, or excising a leaflet on the first or second leaf with a pair of scissors contaminated with the inoculum (Gitaitis et al. 1989). Leaf margin curling and one‐sided wilting or withering of the leaves in the vicinity of the inoculation site will occur within 2–3 weeks, which is indicative of the virulence of the corresponding strain. For artificial inoculation of tomato plants, defoliation using infected scissors and inserting a sterile dissecting needle dipped into a freshly prepared bacterial suspension are more successful than planting in soil containing contaminated plant debris (Kawaguchi and Tanina 2014; Ansari et al. 2019). A paintbrush could be applied to inoculate the surface of small fruits (Medina‐Mora et al. 2001). Disease symptoms become evident from the third day. As the disease progresses, the bacterial population increases in planta, reaching the highest level after 6 days (Tsitsekian et al. 2021). The bacterial concentration can increase to over 10^6^ cells/g plant tissue at 20 cm away from the inoculated point on the stem by 10 days after inoculation.

### 11.3 DNA Sequence‐Based Methods

#### 11.3.1 Conventional PCR

For the first time, Ghedini and Fiore (1995) used a PCR test developed by Sousa Santos et al. (1997) to detect latent *C. michiganensis* infections in tomato seedlings. The sensitivity threshold of the method was estimated around 1.1 × 10^3^ CFU/5 μL of stem suspension (Sousa Santos et al. 1997). Then, specific PCR primers PSA‐4/PSA‐R (Table 1) were developed for the detection of the pathogen along with the other species of the genus (at that time subspecies of *C. michiganensis* sensu lato; Pastrik and Rainey 1997). The applicability of the latter primer pair was improved via multiplexing with tomato‐specific primers NS‐7‐F/NS8‐R as an internal PCR control primer, to provide a reliable method for the detection of *C. michiganensis* (Zhang et al. 2009). Thapa et al. (2020) also developed a multiplex PCR‐based diagnostic platform using the sequences of chromosomal genes *rhuM* and *tomA* and an internal control to amplify both bacterial and plant DNA. Several other PCR primers are available for the specific detection of *C. michiganensis* as detailed in Table 1. Viable cells of the pathogen can be detected using dyes such as ethidium monoazide (Luo et al. 2008) and propidium monoazide (Han et al. 2018) in qPCR tests.

**TABLE 1:** Nucleotide sequences and physical parameters of primers designed for detection and identification of *Clavibacter michiganensis* using conventional PCR, TaqMan‐PCR and LAMP techniques.

Primer name	Sequence (5′–3′)	Size of amplicon (bp)	Annealing temperature (°C)	Target	Reference
**PCR primers for phylogenetic analyses of *C. michiganensis* **					
fD1	AGAGTTTGATCCTGGCTCAG	1484	63	16S rDNA	Weisburg et al. (1991)
rP2	ACGGCTACCTTGTTACGACTT				
atpD2F	GACATCGAGTTCCCGCAC	1104	55	*atpD*	Jacques et al. (2012)
atpD2R	CGATGATCTCCTGGAGCTCCTTGT				
dnakF	GCTCGTGCAGTAGGAATCG	704	59	*dnaK*	Jacques et al. (2012)
dnakR	CTTGGCGATCTGTCGTTCGAGAC				
2F	ACCGTCGAGTTCGACTACGA	977	57	*gyrB*	Richert et al. (2005)
6R	AGSACGATCTTGTGGTA				
ppkF	GAGAACCTCATCCAGGCCCT	604	60	*ppk*	Jacques et al. (2012)
ppkR	CGAGCTTGCAGTGGGTCTTGAG				
recaF	GACCGCGCTCGCACAGATCGACCG	724	63	*recA*	Jacques et al. (2012)
recaR	GCCATCTTGTTCTTGGACGACCTTG				
3Fs	GACAACTTCTACTTCAAC	447	57	*rpoB*	Richert et al. (2007)
4Rs	GTTGTTCTGGTCCATGAAC				
**PCR primers for detection of pathogenicity determinant genes in *C. michiganensis* **					
cel‐578up	ATGGCTTCCCTACGATCC	2193	59	*celA*	Jahr et al. (2000)
cel‐2752low	ACAGGGTAGAAGCGGGAGG				
pCRcel‐593	TCCTTATATGACATTTCGCC	1268	57	Catalytic domain of *celA*	Jahr et al. (2000)
pCRcel‐1860	GCCACTTCGCTGATACAG				
PFC3	GGTACGAAGTTCGAGACGAC	552	62	Cellulose binding domain of *celA*	Kleitman et al. (2008)
PFC5	TGTAGCGGTGAGTCGTGGTGA				
tomA‐F	CGAACTCGACCAGGTTCTCG	529	60	*tomA*	Kleitman et al. (2008)
tomA‐R	GGTCTCACGATCGGATCC				
ppaA‐F	CATGATATTGGTGGGGAAAG	588	56	*ppaA*	Kleitman et al. (2008)
ppaA‐R	CCCCGTCTTTGCAAGACC				
chpC‐F	GCTCTTGGGCTAATGGCCG	639	62	*chpC*	Kleitman et al. (2008)
chpC‐R	GTCAGTTGTGGAAGATGCTG				
chpG‐F	GACAACATGACCCTGCACTG	394	62	*chpG*	Kleitman et al. (2008)
chpG‐R	TCGGGGTGTAGACAAGGAAG				
Cmm‐5	GCGAATAAGCCCATATCAA	609	55	*pat‐1*	Dreier et al. (1995)
Cmm‐6	CGTCAGGAGGTCGCTAATA				
**Generic PCR primers for *Clavibacter* spp**.					
CMR16F1	GTGATGTCAGAGCTTCCTCTGGCGGAT	1425	62	*Clavibacter* spp.	Lee et al. (1997)
CMR16R1	GTACGGCTACCTTGTTACGACTTAGT				
PSA‐4	TCATTGGTCAATTCTGTCTCCC	271	58	*Clavibacter* spp.	Pastrik and Rainey (1999)
PSA‐R	TACTGAGATGTTTCACTTCCCC				
Clav‐F	TGGATCACCTCCTTTCTAAG	296	55.9	rRNA region	Quintero‐Vásquez et al. (2013)
Clav‐R	CACCACCATCCACAACAGGA				
**Specific PCR primers for detection of *C. michiganensis* **					
ITSYG‐1	CGCGTCAGGCGTCTGTT	270	60	rRNA internal transcribed spacer (ITS)	Zhao et al. (2007)
ITSYG‐2	AGTGGACGCGAGCATC				
Probe	TGGCGGTGGCGCTCATGG*				
Cmm141F	CAGGCGTCCGTCGGTGAGGTGGTC				Cho et al. (2012)
Cmm141R	GCGGGAGAGCGGTGCGGGAATG	141	63	Ferredoxin reductase	
Cmm1F	GACAAGCACCTCTACACCTGG	500	69	Tomatinase	Kokošková et al. (2010)
Cmm1R	TTGATCCCTGACTTCAGCGT				
Cmm‐digF	TCTGGGTGTGTCTGGTTTCTTG	61		16S–23S rRNA	Morcia et al. (2023)
Cmm‐digR2	CCCCACCACCATCCACAA				
Cmm‐Pr	FAM‐CGGACCCTTTCCGTCGT‐MGB				
micALAMP2‐F3	CGACAACAGGAACACAGGT	NS	NS		Yasuhara‐Bell et al. (2013)
micALAMP2‐B3	GCCACATTCGATGGTGAGC				
micALAMP2‐FIP	GAGCAGCATGTCCCACCGGGACACGATGAACGACATCCTC				
micALAMP2‐BIP	CGTCCGTCCAGACCCAGATCGCTGGACATGTACGGGCTCA				
micALAMP2‐LoopF	TGACCATGACGGGGGTCT				
micALAMP2‐LoopF probe	/56‐FAM/ACGCTGAGGACCCGGATGCGAATGCGGATGCGGATGCCGATGACCATGACGGGGGTCT				
Quencher probe	TCGGCATCCGCATCCGCATTCGCATCCGGGTCCTCAGCGT/3BHQ_1/				
TomA‐F1	ATGAAGAGCTTCGCGTCCG	630	NS	*tomA*	Thapa et al. (2020)
TomA‐F2	GAGAACACTGACATCCGCAG				
Cm_tomA_F	CGATCCTTCCGTCGTAAC	125	NS	*tomA*	Brochu, Dumonceaux, et al. (2023)
Cm_tomA_R	CCATGGTCTGATCTCCAG				
Cm_tomA_P	TGAAGTGCTCTGTCATCGCCG				
Cm_rhuM_F	GTCGAATAGGAGGAAGCC	149	NS	*rhuM*	Brochu, Dumonceaux, et al. (2023)
Cm_rhuM_R	CGAAGAACTACCTCACCG				
Cm_rhuM_P	TTGAACTTGCTCACCACGAGATTCC				
RhuM‐F	GGGTCGGTTCATCCTGTA	1000	NS	*rhuM*	Thapa et al. (2020)
RhuM‐R	CTTCGGGAGGTTCTCCTGT				
CMM‐16‐23S_e_fwd	GCACCTTCTGGGTGTGTCTG	140	Multiplex	16S–23S rRNA	Peňázová et al. (2020)
CMM‐16‐23S_e_rev	TGTGATCCACCGGAAAACCG				
CMM TP	TCCGTCGTCCTGTTGTGGATG(HEX‐BHQ1)				
CM3	CCTCGTGAGTGCCGGGAACGTATCC	639	60	Chromosomal DNA	Sousa Santos et al. (1997)
CM4	CCACGGTGGTTGATGCTCGCGAGAT				
CmmG‐F_wf	CGTCGAGAACCAGCTCATCA	136	65	*clvG*	Ramachandran et al. (2021)
CmmG‐R_wf	CGAGATGACGGCGTAGTACC				

Abbreviation: NS, not specified.

* The 5′‐end labelled with 6‐carboxyfluorescein (FAM) and the 3′‐end labelled with tetramethycarboxyrhodamine (TAMRA).

#### 11.3.2 Bio‐PCR

Bio‐PCR is an improved derivative of conventional PCR where bacterial cells are enriched on a general or semiselective medium before DNA amplification. Advantages of bio‐PCR over conventional PCR techniques include the elimination of false positives resulting from the presence of dead cells that may be present in the seed, elimination of false negatives due to potential PCR inhibitors in seed extracts, increased sensitivity of detection and no need for DNA extraction prior to amplification (Schaad et al. 1995). Hadas et al. (2005) noted that in tomato seed samples containing 5–10 infected seeds per 10,000 seeds, *C. michiganensis* was detected by agar plating assay on three media (CNS, mSCM and D2ANX) as well as using direct PCR from seeds and bio‐PCR. However, in samples of one infected seed per 10,000 seeds, the pathogen could be detected only by bio‐PCR.

#### 11.3.3 TaqMan‐PCR

Besides conventional PCRs, real‐time TaqMan‐based methods are also available for the detection of the pathogen. In some cases, the PCR primers are capable of targeting all *Clavibacter* species in a single run. For instance, a real‐time TaqMan‐PCR assay was developed for the detection, differentiation and absolute quantification of *C. michiganensis* by directing the amplification of a 223 bp DNA fragment of intergenic sequences of the rRNA operon (ITS) (Bach et al. 2003). Then, a TaqMan probe was developed for the specific detection of *C. michiganensis* in symptomless tomato seeds. The pathogen can be detected in 2 h without DNA extraction when the seed infection rate is higher than 1% (Zhao et al. 2007). The primer pair CmmG‐F_wf/CmmG‐R_wf was designed to detect the *clvG* gene sequence, which is exclusively present in *C. michiganensis* . General *Clavibacter* primers and a universal internal control were also added to neutralise PCR inhibitors and false‐negative results in real‐time PCRs, making a reliable triplex TaqMan qPCR. The assay was specific for *C. michiganensis* and detected up to 10 fg of the pathogen's DNA (Ramachandran et al. 2021). Recently, Brochu, Dumonceaux, et al. (2023) developed a multiplex TaqMan real‐time PCR assay to detect *C. michiganensis* based on two chromosomal virulence‐related genes *rhuM* and *tomA* (Brochu, Dumonceaux, et al. 2023). The plant internal control *tubulin α3* was included in each of the multiplexes to improve the reliability of the assay.

#### 11.3.4 Loop‐Mediated Isothermal Amplification

Loop‐mediated isothermal amplification (LAMP) assay has advantages over immunodiagnostic methods and PCR‐based techniques because of its specificity and isothermal nature, which allows for easy field application. Yasuhara‐Bell et al. (2013) developed a LAMP technique based on the michiganin A (*micA*) gene, which was highly specific to the bacterial canker pathogen. In another study, genomic analyses showed that *clvA*, *clvF* and *clvG* were present only in *C. michiganensis* , not in other *Clavibacter* species nor other genera of plant‐associated bacteria. Thus, loop‐mediated amplification of *clvA* was as effective in identifying *C. michiganensis* ‐positive tomato seed and tissue samples as the ImmunoStrip method (Yasuhara‐Bell et al. 2014). Yasuhara‐Bell et al. (2015) showed that the LAMP technique can provide a reliable real‐time portable in‐field assay comparable to accepted standards. Dobhal et al. (2019) developed another LAMP assay where the sensitivity of the method was 1 fg DNA per reaction.

A comparative in silico analysis of available PCR primers for their specificity to *C. michiganensis* using whole genome resources of *Clavibacter* species is illustrated in Figure 4. The primer pairs CMR16F1/CMR16R1, PSA‐4/PSA‐R and Clav‐F/Clav‐R are generic for all *Clavibacter* species (Lee et al. 1997; Quintero‐Vásquez et al. 2013). The primer pairs Cmm141F/Cmm141R (targeting *ferredoxin reductase*), Cmm1F/Cmm1R (targeting *tomatinase*), RhuM‐F/RhuM‐R (targeting *rhuM*) and CM3/CM4 are highly specific for *C. michiganensis* , where no in silico DNA amplification was observed in non‐ *C. michiganensis* strains even if the strain was isolated from a tomato plant (Cho et al. 2012; Kokošková et al. 2010; Thapa et al. 2020; Sousa Santos et al. 1997). Interestingly, PCR primers originally designed for detection and amplification of pathogenicity determinant genes in *C. michiganensis* have been shown to be entirely specific for this species, where they could amplify the expected DNA fragment only in tomato‐pathogenic strains. Thus, the primers cel‐578up/cel‐2752low (*celA*), PFC3/PFC5 and pCRcel‐593/pCRcel‐1860 (catalytic domain of *celA*), tomA‐F/tomA‐R, Cm_tomA_F/Cm_tomA_R and TomA‐F1/TomA‐F2 (*tomA*), chpC‐F/chpC‐R (*chpC*) and chpG‐F/chpG‐R (*chpG*) are exclusively specific for *C. michiganensis* . The potential applicability of the latter primer pairs for detection and identification of *C. michiganensis* needs to be evaluated in cross‐laboratory assays.

### 11.4 Other Techniques

Amplification and sequencing of the *gyrB* gene using a single primer set has sufficient resolution and specificity to identify each *Clavibacter* species (Richert et al. 2005). However, MLSA/MLST using the sequences of six housekeeping genes (*atpD*, *dnaK*, *gyrB*, *ppK*, *recA* and *rpoB*) is more useful for precise identification, taxonomic delineation, typing and phylogenetic characterisation of *C. michiganensis* populations (Jacques et al. 2012). All species of the genus generate distinct and reproducible matrix‐assisted laser desorption/ionisation time‐of‐flight (MALDI‐TOF) mass spectrometry profiles, with unique and specific ion peaks for each species, which could be used as biomarkers for identification of the bacteria (Zaluga et al. 2011). Early detection of the disease is achievable using machine‐learning spectral analysis (Vallejo‐Pérez et al. 2021). Raman spectra were obtained from asymptomatic *C. michiganensis* ‐infected tomato plants as well as healthy controls with a 785 nm excitation laser micro‐Raman spectrometer. The Raman spectra obtained from infected tomato leaf samples exhibited peaks associated with cellular components (carbohydrates, carotenoids, chlorophyll and phenolic compounds). Raman bands associated with triterpenoids and flavonoids compounds can be considered indicators of *C. michiganensis* infection during the asymptomatic stage (Vallejo‐Pérez et al. 2021).

### 11.5 Simultaneous Detection With Other Pathogens

Simultaneous detection of *C. michiganensis* with other tomato pathogens would save cost and effort in field surveys and quarantine assays. Multiplex PCR provides rapid and low‐cost results for detection of bacterial pathogens of tomato. In some cases, however, the sensitivity of detection may be reduced in simultaneous detections. Özdemir (2009a) developed a multiplex PCR test for simultaneous detection of three seedborne tomato bacterial pathogens, *C. michiganensis* , *Pseudomonas syringae* pv. *tomato* and *Xanthomonas euvesicatoria* pv. *euvesicatoria*. Another multiplex real‐time PCR method based on fluorescent TaqMan probes was developed for simultaneous detection of *C. michiganensis* , *P. syringae* pv. *tomato* and leaf spot‐causing xanthomonads (Peňázová et al. 2020). Besides bacterial canker, pathovars of *X. euvesicatoria* , *P. syringae* and complex species are the main bacterial pathogens infecting tomato with leaf spot, leaf speck and blight symptoms (Osdaghi et al. 2021). Simultaneous detection of *C. michiganensis* and pepino mosaic virus in tomato seed was also reported by Johnson and Walcott (2012). A multiplex PCR assay was developed for simultaneous detection of *C. michiganensis* , *Fusarium* sp., *Leveillula taurica* and begomoviruses (Quintero‐Vásquez et al. 2013). A polyprobe (poly‐3) was developed for simultaneous detection of *C. michiganensis* , pepino mosaic virus and Mexican papita viroid in tomato plants by non‐isotopic molecular hybridisation, which was comparable with real‐time PCR results (Zamora‐Macorra et al. 2015). A chip digital PCR was also developed to identify and quantify *C. michiganensis* and *Ralstonia solanacearum* at the same time (Morcia et al. 2023).

## 12 Management of the Disease

Due to the lack of effective bactericides, the bacterial canker disease is managed primarily by quarantine measures, use of pathogen‐free plant materials and sanitation. A number of nonspecific chemicals, biological control agents and semi‐resistant/tolerant cultivars are also available to keep the established diseases under the economic loss threshold. Quarantine inspection and early detection of the pathogen are the most effective approaches for bacterial canker management in areas with no history of the diseases.

### 12.1 Field Management

Blank et al. (2016) noted that the adoption of field management strategies is the most influential factor on bacterial canker severity. Differences in farmers' experience, differences in agricultural practices between growers and the quality of implementation of management practices are correlated with the occurrence and severity of the disease. The bacterial canker agent is capable of being epiphytic on plants. All actions allowing contact between plant surfaces or between plant sap should be avoided. Touching symptomless infected plants bearing guttation droplets prior to touching nearby plants spreads the pathogen over distances within rows of up to 22 plants (Sharabani, Manulis‐Sasson, et al. 2013; Sharabani, Shtienberg, et al. 2013). Infection is transferred to healthy plants by cutting with contaminated scissors after cutting infected plants with early symptoms or symptomless ones (Sharabani, Manulis‐Sasson, et al. 2013; Sharabani, Shtienberg, et al. 2013). As the bacterium survives easily in water, irrigation should be operated with great caution. The sub‐irrigation system reduces, but does not prevent, pathogen dispersal. Jones, Worobo, et al. (2014) proposed UV light inactivation of *C. michiganensis* in unfiltered surface irrigation water where > 99.9% inactivation was achieved. Frenkel et al. (2016) showed that the pathogen dispersed spatially from root‐inoculated source seedlings and colonised the leaf surfaces of surrounding seedlings to distances of 65–75 cm.

### 12.2 Biological Control

While Proteobacteria are the most abundant organisms within the endophytic communities of diseased tomato (López et al. 2020), *Pseudomonas* spp., *Streptomyces* spp. and *Bacillus* spp. are considered the most appealing biological control agents for *C. michiganensis* worldwide (Aksoy et al. 2017; Benchlih et al. 2023). Fluorescent pseudomonads isolated either from the phyllosphere or rhizosphere of tomato have frequently been reported as antagonistic agents against the incidence of bacterial canker (Boudyach et al. 2001; Amkraz et al. 2010; Lanteigne et al. 2012; Bouizgarne et al. 2023). A rhizosphere strain of *Pseudomonas entomophila* (23S) was reported to have a strong antagonistic activity from which two anti‐ *C. michiganensis* compounds, C15H16N2O and C16H18N2O, were isolated (Takishita et al. 2021). Paulin et al. (2017) demonstrated that inoculation of tomato plants with 2,4‐diacetylphloroglucinol‐ and hydrogen cyanide‐producing *Pseudomonas brassicacearum* LBUM300 could significantly reduce bacterial canker symptoms.

The gram‐positive bacteria Bacilli and Actinobacteria have also a significant role in the biological control of *C. michiganensis* (Utkhede and Koch 2004; Zhang et al. 2010). Calderón‐de la Sancha et al. (2022) reported the antimicrobial activity in a low‐molecular‐weight protein secreted naturally by *Streptomyces lividans* TK24 when glucose or glycerol were used as carbon sources. Water extracts of *Bacillus* sp. strains H8‐1 and K203 inhibited wilting caused by *C. michiganensis* and slowed the pathogenic colonisation in tomato plants. The relative expressions of *celA*, *celB*, *pat1* and *pelA* of *C. michiganensis* treated with the bacterial aqueous extracts were reduced compared to controls at 72 h after treatments (Jang et al. 2022). *Bacillus* strains 1B‐23 and 1D‐12, capable of producing surfactins A, B and C, significantly reduced disease incidence in a greenhouse setting (Laird et al. 2020).

Application of *Pseudozyma aphidis* spores on tomato plants in greenhouses significantly reduced incidence of bacterial canker disease. *P*. *aphidis* activates PR1a and other pathogenesis‐related genes in tomato plants and can trigger an induced resistance response against *C. michiganensis* that proceeds in a salicylic‐acid (SA)‐independent manner (Barda et al. 2015). Exogenously applied SA suppressed bacterial growth and induced the expression of WRKY transcription factors, suggesting that some *C. michiganensis* ‐responsive genes are regulated by SA signalling and SA signalling activation should improve tomato immunity against *C. michiganensis* (Yokotani et al. 2021). Enzymatic activity, hydrogen peroxide formation and lignin production were significantly higher in benzothiadiazole (500 ppm)‐treated leaves than in those observed in the control (Tripathi et al. 2022).

Promising results have also been reported on phage therapy of the bacterial canker disease. Application of Phage33 from tomato fields in Turkey was effective against *C. michiganensis* (Bekircan Eski and Darcan 2023). Bacteriophage CMP1 (*Siphoviridae* family) infects *C. michiganensis* specifically, encoding a peptidase that was shown to effectively lyse the pathogen specifically (Wittmann et al. 2011). The endolysin gene of CMP1 was transferred into tomato plants by *Agrobacterium*‐mediated transformation, where transgenic tomato plants did not show disease symptoms after infection with *C. michiganensis* (Wittmann et al. 2010, 2016).

Preliminary investigations revealed suppression of *C. michiganensis* by plant extracts and essential oils while the applicability and mass production of these biological agents on a large scale are still questionable. Aqueous extract of *Eucalyptus globulus* leaves inhibits the growth of *P. syringae* pv. *tomato*, *X. euvesicatoria* pv. *euvesicatoria* and *C. michiganensis* (Pinto et al. 2023). Capsaicinoids are molecules found in the fruits of *Capsicum* species that produce their spicy taste and also possess antibacterial and antifungal effects. A synthetic capsaicinoid oleoresin showed an inhibitory effect against *Fusarium oxysporum* and *C. michiganensis* (Valencia‐Hernandez et al. 2022). Sonicated extracts from microalgae of the genera *Leptolyngbya* and *Scenedesmus* were evaluated for their effect on bacterial canker inhibition. Bioassays on tomato seedlings showed that root application of *Scenedesmus* extract is capable of controlling *C. michiganensis* , while foliar and root application of *Leptolyngbya* extract seems to be more related to the strengthening of the plant through the SA route (Toribio et al. 2021). Growth and oxygen consumption of *C. michiganensis* were suppressed after the addition of fragarin to cultures. Fragarin is an antibiotic that was isolated and purified from a soluble fraction of strawberry leaves. Furthermore, dissipation of the membrane potential and an increase in cell membrane permeability were observed in the presence of fragarin (Filippone et al. 2001).

### 12.3 Chemical Control

Chemical control with cupric bactericides or streptomycin is the last defensive line in canker disease management (Lamichhane et al. 2018; Lyu et al. 2019). To prevent severe bacterial canker disease in the field, growers should initiate and sustain bactericide applications to tomato transplants in the greenhouse to suppress pathogen populations. de León et al. (2008) showed that treatments containing copper sulphate greatly reduced disease symptoms on plants, while streptomycin was less effective. Coskun and Horuz (2023) noted that foliar spray of phosphites inhibited the growth of *C. michiganensis* between 50% and 74% and raised the chlorophyll concentration of tomato leaves up to 30% in phosphite‐sprayed plants. Besides traditional copper‐based chemicals and antibiotics, application of novel bactericides, for example, nanoparticles, provides new tools for management of the disease (Marcelino‐Pérez et al. 2021). Silver nanoparticles (AgNPs) are promising inhibitors of *C. michiganensis* (Rivas‐Cáceres et al. 2018). Application of copper nanoparticles and potassium silicate was effective in reducing the severity of *C. michiganensis* (Cumplido‐Nájera et al. 2019). AgNPs were prepared from an aqueous extract of fresh leaves from *Larrea tridentata* , with significant disease control being achieved 42 days post‐inoculation with the pathogen (Méndez‐Andrade et al. 2022). Furthermore, AgNPs produced with moringa extracts reduced canker disease by 86%. Systemic acquired resistance is suggested as an important mechanism induced by Mo‐AgNPs (Mercado‐Meza et al. 2023).

Pretreatment of plants with acibenzolar‐*S*‐methyl (benzo [1,2,3]thiadiazole‐7‐carbothioic acid‐*S*‐methyl ester, ASM; Bion 50 WG) reduced the severity of the disease as well as the growth of the bacteria in planta (Soylu et al. 2003; Baysal et al. 2003). Although in vitro growth of the bacteria was not affected by dl‐β‐amino butyric acid (BABA) treatment, foliage sprays of 500 μg/mL BABA significantly suppressed disease development up to 54% by day 14 after inoculation (Baysal et al. 2005). Polygodial and nordrimenone showed promising results against *C. michiganensis* , *P. syringae* pv. *tomato*, *F. oxysporum* f. sp. *lycopersici* and *Phytophthora* spp. (Xu et al. 2015; Montenegro et al. 2018). The synthetic elicitors 2,6‐dichloro‐isonicotinic acid (INA) and 2,4‐dichloro‐6‐{(*E*)‐[(3‐methoxyphenyl)imino]methyl}phenol (DPMP) enhance tomato resistance against bacterial canker disease with different molecular mechanisms (Bektas 2021). Phenolic and flavonoid contents of medicinal plants belonging to 16 species were approved against the tomato bacterial canker agent (Amkraz et al. 2014). Ombiro et al. (2018) reported that ralhibitin E completely inhibited the growth of *C. michiganensis* and *R. solanacearum* at 10 μg/mL. Eustressic doses of cadmium (60 μg/kg of soil) induce defence mechanisms and protection against *C. michiganensis* in tomato (Valencia‐Hernandez et al. 2023). Rotondo et al. (2023) introduced a proprietary blend of plant extracts as a potential option for bacterial canker management and yield enhancement in hydroponic tomato greenhouse production systems.

#### 12.3.1 Antibiotics

Applications of copper hydroxide, copper hydroxide+mancozeb, copper hydroxide+mancozeb (premixed 12 h before spraying), streptomycin and streptomycin+copper hydroxide to seedlings in the greenhouse increased the survival of inoculated transplants in the field in comparison to the control (Hausbeck et al. 2000). Resistance to streptomycin was reported in *C. michiganensis* strains isolated in Chile (Valenzuela et al. 2019). Minimum inhibitory concentration in a naturally occurring streptomycin‐resistant *C. michiganensis* strain TX‐0702 was 128 μg/mL (Lyu et al. 2019).

#### 12.3.2 Seed Treatment

Citric acid at 0.1 M concentration has been proved to be useful for the elimination of *C. michiganensis* from tomato seeds (Özdemir 2009b). HCl was used to treat the tomato pulp in seed extraction. This treatment, followed by drying the seeds for 3 h, achieved pathogen eradication (Thyr et al. 1973). In contrast, acid extraction by soaking pulp in an equal volume of 5% HCl for 10 min, followed by washing, did not entirely eliminate *C. michiganensis* from naturally infected seeds (Pradhanang and Collier 2009). Ten‐minute immersion of seeds in acidified nitrite resulted in 98% of the seeds being pathogen‐free. Treatment with copper hydroxide and certain strains of *Bacillus* spp. resulted in 100% pathogen‐free seeds (Kasselaki et al. 2011).

## 13 Host Resistance

### 13.1 Resistance Sources

Development of resistant cultivars is the most sustainable approach for long‐term management of the bacterial canker diseases as well as for all seedborne bacterial pathogens of the crop (Osdaghi et al. 2021; Khojasteh et al. 2024). Screening and breeding practices for disease resistance will be more applicable when they are conducted simultaneously for a set of economically important diseases. The genetic nature of disease resistance is classified as either being qualitative (simple), controlled by one or few resistance genes, or quantitative (complex), governed by multiple resistance genes or quantitative trait loci (QTLs). Meta‐analysis of QTLs, which gathers QTL data from independent studies across genetic backgrounds and environments and identifies stable and reliable QTL regions, is a powerful strategy to facilitate marker‐assisted selection in plant breeding. Recently, Khojasteh et al. (2024) examined 491 QTLs previously reported for resistance to tomato diseases in 40 independent studies and 54 unique mapping populations. They identified 29 and 44 meta‐QTLs for resistance to bacterial and fungal pathogens, respectively. Among the 29 meta‐QTLs identified for resistance to bacterial diseases, 12, 4 and 2 were specific to bacterial spot, bacterial wilt and bacterial canker diseases, respectively. Interestingly, four meta‐QTLs located on chromosomes 4 and 6 contributed to resistance to both bacterial spot and bacterial wilt diseases, while two meta‐QTLs located on chromosomes 2 and 5 conferred resistance to both bacterial canker and bacterial spot diseases. Abebe et al. (2022) conducted a QTL‐Seq analysis for identification of resistance loci to bacterial canker in tomato. A genomic region (37.24–41.15 Mb) associated with bacterial canker resistance on chromosome 6 (*Rcm6*) was found. Celik (2023) constructed a physical map for bacterial canker resistance QTLs and identified QTL‐specific candidate genes. Single‐marker QTL analysis suggested that at least two loci originating from *Solanum hirsutum* LA407, Rcm 2.0 on chromosome 2 and Rcm 5.1 on chromosome 5, contribute to resistance in replicated trials (Kabelka et al. 2002).

While some cherry tomato varieties are tolerant, others are highly susceptible to bacterial canker. In general, cherry tomato varieties tend to be more resistant to bacterial canker but more susceptible to bacterial spot than the fresh‐market tomato (Romero et al. 2003). The pathogen causes less severe symptoms in wild tomato species (e.g., *Solanum habrochaites* LA2128, *Solanum arcanum* LA2157 and *S. arcanum* LA2172) and is impeded in spread and colonisation of the vascular system (Peritore‐Galve et al. 2020). QTLs conferring tolerance in *S. arcanum* and *S. habrochaites* have been identified (Peritore‐Galve et al. 2020). Using RFLP markers by means of the Kruskal–Wallis rank‐sum test in *Solanum peruvianum* accession LA2157, five regions on chromosomes 1, 6, 7, 8 and 10 were identified that may be involved in *C. michiganensis* resistance (Sandbrink et al. 1995; Van Heusden et al. 1999). Significant variation in disease resistance was observed among 283 somaclones from 12 tomato cultivars (De Vries and Stephens 1997). Francis et al. (2001) noted that partial resistance to *C. michiganensis* was identified in a wild relative of cultivated tomato, *S. habrochaites* LA407.

### 13.2 Host Responses During Tomato– *C. michiganensis* Interactions

Upon *C. michiganensis* inoculation, several defence responsive genes were found to be differentially expressed, of which 26 genes were in the resistant line and three were in the susceptible line (Basim et al. 2021). Advanced lines of a cross between *S. arcanum* LA2157 and *S. lycopersicum* showed that introgression lines carrying a locus of *S. arcanum* LA2157 on chromosome 7 had high levels of tolerance to *C. michiganensis* . Koseoglou, Brouwer, et al. (2023) suggested that two additional loci on chromosomes 2 and 4 together with the locus on chromosome 7 are required for tolerance to *C. michiganensis* . In RNA‐seq analyses, 1788 and 540 genes were up‐regulated and down‐regulated upon infection with *C. michiganensis* , respectively, where genes involved in the defence response, phosphorylation and hormone signalling were over‐represented. Tomato genes involved in SA and phenylalanine ammonia‐lyase (PAL) pathway were also up‐regulated upon infection (Yokotani et al. 2021). Koseoglou, Hanika, et al. (2023) showed that tomato gene *SlWAT1* is a susceptibility gene to *C. michiganensis* , where inactivation of this gene leads to reduced susceptibility to the bacterium, reduced free auxin content and ethylene synthesis in tomato stems. Host‐derived ethylene plays an important role in the regulation of the tomato susceptible response to *C. michiganensis* (Balaji et al. 2008). Comparative transcriptome and gene ontology analysis revealed that the wall‐associated receptor kinase‐like 20 (WAKL20) contributes to resistance against *C. michiganensis* infection (Deng et al. 2023).

### 13.3 Screening for Resistance

No immunity has been found in any tomato cultivars against *C. michiganensis* . Berry et al. (1989) demonstrated that it is possible to identify plants with intermediate resistance using a dilute inoculum of a virulent strain of *C. michiganensis* . Sen et al. (2013) screened 24 different wild accessions of tomato and found several new tolerant sources: *Solanum pimpinellifolium* GI.1554, *Solanum parviflorum* LA735 and *S. parviflorum* LA2072. They also confirmed the tolerance in *S. peruvianum* LA2157, *S. peruvianum* PI127829, *S. peruvianum* LA385, *S. habrochaites* LA407 and *S. lycopersicum* IRAT L3. A new method using the PathoScreen field test kit was evaluated to localise green fluorescent protein‐tagged *C. michiganensis* in planta and to quantify the pathogen based on the percentage of corrected GFP (cGFP%). The system was sensitive in detecting the GFP‐tagged *C. michiganensis* in the shoots, but in the roots, a high autofluorescence masked detection and thus sensitivity of the assay (Mohd Nadzir et al. 2019). Brochu, Durivage, et al. (2023) aimed to identify an artificial inoculation method to induce bacterial canker on tomato plants in greenhouse conditions to homogenise the results of different studies. The syringe inoculation with low fertilisation was the most effective inoculation method, allowing the development of a multilevel scale that can be used to study the interaction between tomato plants and *C. michiganensis* . Excision with an infected scalpel of the first true leaf of 3‐week‐old seedlings, followed by applying a drop of inoculum on the wound, discriminated well between populations of partially resistant and susceptible tomato genotypes (Van den Bulk et al. 1991).

## 14 Conclusion and Future Avenues for Research

Bacterial wilt and canker caused by *C. michiganensis* is considered a devastating disease in many tomato production regions. Since the first description of bacterial canker in 1909, dozens of studies provided a foundation of knowledge, that is, understanding of pathogen diversity, colony morphology and factors influencing the pathogen spread. Investigations highlighted the role of quarantine inspections, crop sanitation, and resistant cultivars in the management of bacterial canker disease. The use of pathogen‐free high‐quality seedlots is the cornerstone of disease management in the areas where the pathogen is established. However, strict quarantine rules should prevent the distribution of the pathogen into new areas with no history of the disease. Recent technological advancements in high‐throughput DNA sequencing ensure that in the coming years we will integrate all discoveries into a comprehensive understanding of the biology, spread and survival of *C. michiganensis* , as well as molecular mechanisms underlying disease development in the host plant. During the past two decades, genomics has played an increasing role in the understanding of colonisation, infection, transmission and evolution of plant‐pathogenic bacteria. Comparative genomics analyses and phylogenomics provided fundamentals for elucidating the global population structure of *C. michiganensis* . The first complete genome sequence of *C. michiganensis* became available in 2008, and by early 2024, 265 *C. michiganensis* genome sequences were deposited in the NCBI public database. These whole genome resources provide opportunities to deepen the understanding of the molecular basis for the morphological variations and colony pigmentation of the pathogen. Further, with the genomics data in hand, we will be able to develop state‐of‐the‐art genome‐informed detection methods to detect seed infections with lower efforts and cost. Such knowledge could aid in mitigating the negative impacts of colony variations in *C. michiganensis* in quarantine inspections and seed health tests. This will also enable molecular breeders to develop durable broad‐spectrum resistance and disease control strategies that are acceptable to the tomato industry. Further studies on plant and pathogen transcriptomics and meta‐transcriptomics will initiate a deeper understanding of plant–bacterial interactions and ways of controlling plant colonisation. Finally, recent advances in our understanding of molecular host–pathogen interactions of other plant pathogens in the *Microbacteriaceae* will continue to aid the development of a more comprehensive understanding of the molecular biology of corynebacterial plant pathogens and identify research paths for the sustainable management of bacterial canker in the 21st century.

## Conflicts of Interest

The authors declare no conflicts of interest.

## Data Availability

Data sharing is not applicable to this article as no new data were generated.
